# Game theory-based analysis of policy instrument consequences on energy system actors in a Nordic municipality

**DOI:** 10.1016/j.heliyon.2024.e25822

**Published:** 2024-02-04

**Authors:** Robert Fischer, Andrea Toffolo

**Affiliations:** Energy Engineering, Division of Energy Science, Luleå University of Technology, SE-97187 Luleå, Sweden

**Keywords:** Energy system optimization, game theory, policy instruments, Energy transition, Renewable energy, Carbon emissions, Heating systems

## Abstract

The transition of energy systems requires policy frameworks and instruments to make both energy suppliers and consumers contribute to the common goal of emission reductions and to fairly allocate costs and benefits among market actors and the government. Assuming that market actors – suppliers and consumers adhering to their economic interests – would benefit from cooperating to mitigate emissions, this study applies a game theory-based approach to investigate the interaction between a local electricity supplier and a group of heating consumers not connected to district heating. Selected policy instruments are tested, and their consequences are analyzed in the context of a representative Nordic municipality. The results show that the auction-based Contract for Difference policy instrument is the most suitable one in the studied Nordic context to achieve significant levels of CO2 emissions reduction. It creates a higher level of strategic interaction between the actors, that would be lacking otherwise, under the form of transfer payments from consumers to supplier, and avoids costs to the general taxpayer. While this is sufficient to promote the investments in renewables by the supplier, additional subsidy policies are required to enable the heating consumers to invest in more capital-intensive energy efficiency measures or biomass heating.

## Highlights

• The interaction between a local utility and heating consumers is studied with game theory.
• The benefit of cooperation between private heating consumers and local utilities is minimal.
• Contract for Difference is the policy instrument suitable to achieve local decarbonization.
• It promotes the investment in renewables by the supplier without costs to taxpayers.
• Subsidies are required to reduce the electricity demand from private heating consumers.

## 1 Introduction

In its 6th assessment report, the IPCC concluded that global warming would exceed 1.5 °C and 2 °C unless deep reductions in GHG emissions occur in the coming decades [1]. Correspondingly, in its recent roadmap report "Net-zero by 2050″, the IEA expressed the need for a total transformation of the energy systems [2]. In this context, cities will have an essential role due to their high energy consumption, and especially Nordic cities because of the heating demand related to the sub-arctic climate [3].

Mitigating heating-related emissions will be crucial to achieve climate targets [4]. The building stock not connected to district heating (DH), which represents about 50% of the Nordic heating market, uses direct electric heating or electric boilers, heat pumps (HP), or biomass boilers [3,5]. Electric heating has several issues, including the GHG emissions associated through the grid emission factor, the vulnerability of Swedish households to electricity price variations [5], and the increased risk for municipalities to experience capacity bottlenecks in the existing electric grid infrastructure due to growing electricity demand [6,7].

Local authorities have limited or no control over building standards or installed heating technologies and over emissions related to electricity use. Therefore, national policy instruments must be formulated so that local market actors can act. Such instruments need to be effective in ensuring that all market actors on the supply and the demand side, as well as the society, the government, or the general taxpayer, fairly share both costs and benefits of mitigation and adaptation measures [8,9].

### 1.1 Effective policies and policy instruments

Policy instruments can be categorized in different ways. Market-based instruments utilize market forces to transform the energy system, the major instruments being carbon pricing, support for renewable energy (RE) deployment, and energy efficiency (*EE*) measures. Non-market-based instruments include building codes and vehicle efficiency standards, public investments in RD&D, and mandates, for example, solar water heating or replacement of fossil-fuel heating. Enabling policy instruments, aimed at generating a favorable environment for energy transition, complement the two former categories [10,11].

Carbon pricing mainly refers to two instruments – emission trading schemes and carbon taxation. The European emission trading scheme (EU-ETS) works on the ‘cap and trade’ principle, covering emissions from combustion power and heating plants above a capacity threshold of 20 MWth and other large-scale industries [12]. Carbon taxes in EU member states put a carbon price on the emissions of sectors not covered by the EU-ETS. A carbon tax of at least 30 EUR/tCO2 should be implemented to be effective according to Ref. [13], while other studies suggest much higher levels (e.g., Ref. [14]).

Policy instruments supporting the deployment of RE generation include feed-in-tariffs, tradable certificates, such as the Swedish electricity certificate scheme [15], and auction-based instruments, such as the Contract for Difference scheme in the UK and similar schemes in other countries [16,17]. Challenges with such policies are under- and overcompensation of investors and the fair allocation of costs among consumer sectors, the government, and the general taxpayer [18]. Support policies for *EE* can be designed as subsidy schemes, such as investment grants, soft loans, tax deductions, *EE* obligation schemes, or energy performance contracting [19,20].

Research suggests that the most expedient climate policies are a) a global carbon price including penalizing non-participation, b) support of RD&D of low and no-carbon technologies followed by deployment subsidies in the early stages of commercialization, and c) subsidizing *EE* retrofits of buildings [14].

### 1.2 Energy systems analysis – two major approaches

There are two major approaches to the investigation of transition pathways towards a desirable energy future. Firstly, climate-economy models known as integrated assessment models (IAM) or also the more comprehensive computable general equilibrium models (CGE) explore the interplay of economy, society, energy, and the Earth system [[21], [22], [23]]. These are also considered top-down models, where sectors are aggregated with different levels of detail. Nordhaus's dynamic integrated climate-economy model (DICE) represents a much-cited IAM [14,24]. Another well-known IAM is the WITCH model (A World Induced Technical Change Hybrid Model [25], with applications in Ref. [26]). WITCH combines a top-down CGE model with a detailed bottom-up energy model and captures the central economic interrelationships among world regions. The other major approach is energy system optimization and modeling (ESOM). ESOM is considered a technically detailed bottom-up approach and utilizes linear programming techniques to minimize the system-wide cost of energy investments and can include a single or multiple energy sectors [27,28]. ESOM frameworks, methods, and tools include the well-established MARKAL/TIMES model generators and their family of global, regional, national, and local applications [[29], [30], [31]]. A few others important to mention are Balmorel [32], MESSAGEix [33], PRIMES [34], OSeMOSYS [35,36] and Temoa [33]. Multi-objective optimization approaches solved with evolutionary algorithms complement such ESOM models [37,38].

Ideally, comprehensive climate-economic and energy systems modeling would a) be technologically explicit, as ESOM is, b) consider the interactions among society, economy, energy sectors, environment and climate, as IAM/CGE models do, and c) take into account microeconomic realism such as decision-making by companies and consumers when selecting technologies, as CGE models do by representing real-world responsiveness to policies, however on an aggregated level [39]. The Swedish TIMES-CGE model [40], the WITCH model [25] and the European PRIMES model [34] are examples of soft-linked or hybrid models that intend to address the weaknesses of less comprehensive IAM and ESOM. Challenges, however, often lie in the increased complexity and in keeping those combined models theoretically consistent, empirically valid, and computable [23,41,42]. ESOM often does not or cannot consider the peculiarities of the strategies rationally chosen by different actors, as individual suppliers' and consumers’ profit functions and their mutual interactions are not explicitly modeled [43,44]. IAM and ESOM do not study the strategic interactions between rational decision-makers, where non-cooperation or cooperation can result in substantially different outcomes. The mathematical framework of game theory provides tools to analyze such interactions [[45], [46], [47]].

Game theory-inspired methods have been applied in some niches of the energy sector. For example, the coordination between wind farms and power-to-gas facilities to provide flexibility to electricity systems has been investigated using a cooperative game approach [48]. Benefit allocation among commercial buildings in a DH network has been studied [49]. Another research found lower levelized costs for electric generation expansion planning in deregulated markets [50]. An integrated energy system analysis, including compressed air energy storage, resulted in a higher net present value for the players in a cooperative game compared to the individual suppliers acting alone [51]. Optimal capacities for generation and storage and the market-clearing price were determined in Ref. [52] for a group of microgrids incorporating solar PV, wind power, fuel cells and electric vehicles. It can be concluded that previous game theory-inspired research mainly focused on competing players with similar roles in the energy system, including electricity or heat suppliers or groups of energy consumers.

### 1.3 Research gap and novelty of this paper

On the contrary, it appears that game theory approaches are not applied to a set of actors having different roles within the considered energy system. For them it is rather customary to proceed with a more traditional approach in energy system optimization, i.e. defining a single overall economic objective function (total system cost) to be minimized without taking into account the advantages or disadvantages as seen by each of the actors involved. However, these actors are most often the real decision makers that will choose their strategic actions separately and from their own economic point of view, making a game theory approach essential to better understand the future transition of energy systems.

A previous paper by the present Authors [53] was focused on showing the difference between a traditional “total system cost” approach and a game theory approach in determining the optimal strategies of a supplier and a group of consumers in order to reach a given environmental target on the reduction of CO2 emissions. It was concluded that a game theory approach is more suitable to identify the circumstances influencing the strategies chosen by the actors, realistically considers their interactions and is able to fairly harmonize their economic interests.

The novelty of this paper is represented by a game theory approach, applied to the same problem setting of [53], that is used to investigate the effect of different policy instruments on the optimal strategies chosen by the actors and, in turn, on the resulting CO2 emissions without imposing a constraint on the level of their reduction. This approach makes it possible to discard another less than realistic element in energy system modelling and optimization. In fact, in the real world the achieved reduction of CO2 emissions cannot be the result of an artificial mathematical imposition (as if a policy target could be enforced by decree), but it is rather the combined outcome of the implementation of a policy instrument and the consequent optimal strategies chosen by the actors reacting to it.

To make the research gap and the novelty of this paper clearer, a comparison of the main features of the approaches followed by the literature mentioned in the previous section, by paper [53] and by the present paper is offered in Table 1.

**Table 1:** Features of selected mentioned literature compared.

Ref	Approach	Name, tool	System boundaries/application	Policy/constraints
[14]	IAM	DICE. Optimization implemented in GAMS.	Global social welfare function. Estimating the social cost of carbon.	Constraining temperature increases for some cases. Annual emission reduction rate.
[25]	Hybrid	WITCH. Optimization implemented in GAMS.	Regionally disaggregated global model. Includes CO2 emissions from fossil fuel use and from land use change.	Analyzing mitigation policies; budget and technological constraints. Emission trading.
[32]	Partial equilibrium model	Balmorel. Optimization implemented in GAMS.	Country, regions (within a country), areas (within a region). Electricity sector.	Emission constraints; minimum share of renewables; restrictions on fossil fuel consumption; degree of self-sufficiency.
[33]	IAM	MESSAGEix (IIASA). Optimization implemented in GAMS.	Global. All energy sectors. An open framework for integrated and cross-cutting analysis of energy, climate, the environment, and sustainable development.	Carbon tax, emission limits, technology shares, resource limitations.
[34]	Hybrid	PRIMES. Mixed-integer programming problem with equilibrium constraints.	EU member states. Energy sectors.	Environment policies at the Member State or EU level, including taxation, subsidies, ETS, technology and efficiency promoting policies, and technology standards.
[35]	ESM	OSeMOSYS. GNU Mathprog and GAMS.	Global, continental, countries, regions, and cities. Single or multiple energy sectors.	Emission limits, renewable energy targets.
[37,38]	ESM	MOEA and EnergyPLAN	Country, region (state), city. Electricity, heating, transport. Minimizing emissions and costs.	Technology constraints.
[48]	ESM	Stochastic programming	Wind farms and Power-To-Gas facilities are the actors.	Emission reduction and renewable energy targets.
[49]	ESM	MILP + GT (benefit allocation)	Local area (a number of buildings are the actors). Heating sector.	Technology constraints.
[50]	ESM	MOOP + GT	Country. Electricity sector. Renewable power generation as actors.	Technology constraints.
[51]	ESM	Multi-agent optimization + GT	Four plants: solar PV, wind, CHP, and compressed air energy storage (CAES), wherein plant operators act as players.	Technology constraints.
[52]	ESM	Stochastic + GT	Microgrid with wind, solar PV, fuel cells, electrolyzer, battery, and microturbine as actors.	Technology constraints.
[53]	ESM	MOEA + GT	Municipality. Electricity sector and parts of the heating sector.	- Emission targets - Investment subsidies for energy efficiency
**This paper**	ESM	MOEA + GT	Municipality. Electricity sector and parts of the heating sector.	- No emission constraints - CO2-tax - Electricity certificate - Contract for difference - Investment subsidies for energy efficiency and biomass boilers

### 1.4 Aim and scope of this paper

**Fig. 1:**
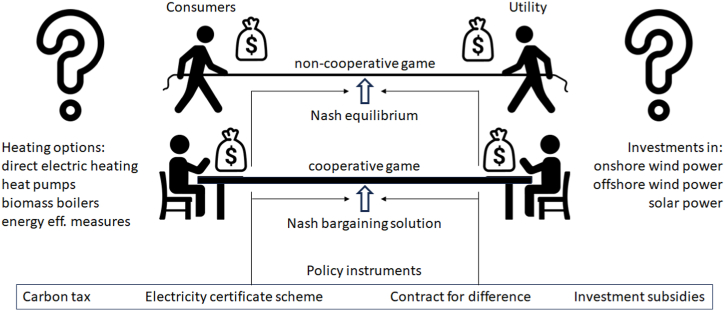
Graphic illustration of the game theory approach to solve the municipal energy system optimization problem, showing the actors, their choices and the implemented policy instruments.

This paper applies a game theory-inspired approach to evaluate the consequences of policy instruments on independent actors with different roles in a Nordic municipal energy system. The representative municipality of Piteå (Sweden) is used as a case study, in which two players are defined – a local electricity supplier and the group of heating consumers not connected to the district heating system. The considered policy instruments are a carbon tax, the Swedish electricity certificate scheme, the auction-based Contract for Difference, and investment subsidies. The possible interactions between the players in the choice of their optimal strategies are analyzed and compared with a non-cooperative and a cooperative approach (see Fig. 1).

In light of the novelty of the approach highlighted in the previous section (i.e. the evaluation of the environmental benefits following the optimal strategies chosen by the actors of the energy system as a reaction to the implemented policy instruments), the research question of this paper is to identify which of the considered policy instruments are more suitable to achieve relevant CO2 emission reductions in the modeled municipal energy system. The general assumptions are that.

- The two considered actors rationally decide their strategy.
- No criterion other than their own economic interest is followed.
- All the other actors in the municipal energy system do not have any strategic choice to react to the implementation of the policy instruments, so they are just affected passively by the consequences of the strategies chosen by the two actors considered here.

The suitability of a policy instrument will also be assessed according to the following conditions.

a) No particular actor shall unfairly be forced to go against its economic interest.
b) Investments (on both sides) shall not be over- or under-compensated.
c) Both suppliers and consumers shall be enabled to contribute to the common goal of emission reductions.
d) Costs for the consumers and the government (the general taxpayer) shall not be unreasonably increased.

The paper first presents a methodology section, describing the game theory-based approach, the municipal energy system modeled in EnergyPLAN and interfaced with a multi-objective optimization algorithm providing Pareto optimal solutions, and the studied policy instruments. Then a section analyzes the results about the investigated policy instruments, which are discussed in a separate section, and, finally, the paper closes with conclusions.

## 2 Method, energy system model, and policy instruments

A game theory-based approach is applied to evaluate the strategies of two market actors in a Nordic context – a local electricity supplier and heating consumers. These actors are part of the municipal energy system of a representative Nordic municipality. Different policy instruments aiming for carbon emission reductions by contributions from both the supplier and the consumers are implemented.

### 2.1 The game theory-based approach

Game theory investigates strategic situations where the outcome measured by one actor depends not only on the own actions, but on the actions of the other actors as well. Every situation between a monopoly and perfect competition is a strategic one. The fundamental game theory concepts used in this paper are briefly introduced in this section. The reader is referred to textbooks on the subject for more details (e.g., Ref. [47]).

Three elements define the context of a game.

- A set of *players*, which represent interacting decision-makers (e.g., competitive firms in imperfect competitions, or suppliers and consumers);
- A set of *strategies* or *actions* available to each player (e.g., choice of technologies and capacities);
- The *preferences* each player has about the own actions and their outcomes, which are measured by individual gain functions, such as profit, net present value or cost reductions by specific investments. These *gains* or *payoffs* depend not only on the own actions, but also on those of the other players.

#### 2.1.1 Non-cooperative game and Nash equilibrium

A non-cooperative game is a game with competition among individual players, who may have perfect knowledge of the strategies available to the other players (in “complete information” games); however, each player does not know, and thus cannot agree upon, which of these strategies will be implemented by the other players [45]. A Nash equilibrium (NE) in a non-cooperative game is an outcome in which no player can increase their payoff by unilaterally modifying the chosen strategy given the strategies chosen by the other players. Such a solution is not necessarily a Pareto optimal outcome of the game, in which no payoff for a player could be improved without reducing the payoff for at least another player. The outcome of a non-cooperative game is fair in the sense that all actors have the best payoff regardless of the strategy chosen by the others.

#### 2.1.2 Cooperative game, disagreement outcome, and Nash bargaining solution

In a cooperative game, the players agree to coordinate their strategies to achieve higher individual payoffs than those obtainable without cooperation. Allocating the higher individual payoffs (or the common surplus) fairly among the players is a surplus-sharing or bargaining problem [46]. The fair allocation, known as the Nash bargaining solution (NBS), is to be identified among the Pareto optimal solutions obtained from a multi-objective optimization problem in which the objective functions are the players’ payoffs. Contrary to NE, the NBS can be proven to exist and be unique. In a cooperative game with only two actors, it is precisely the point *x* that maximizes the following expression (Eq. (1)):

(Eq. 1) (G1(x)‐G1(d))(G2(x)‐G2(d))

where *G1* and *G2* are the gain functions of Player 1 and 2, respectively, and *d* is the disagreement outcome (DO), i.e. the set of strategies the players would adopt without cooperation. Since *G1(d)* and *G2(d)* are the DO payoffs, players act as if they seek to maximize the product of the excess payoffs with respect to the DO. The product of the excess payoffs is generally referred to as the Nash product.

**Fig. 2:**
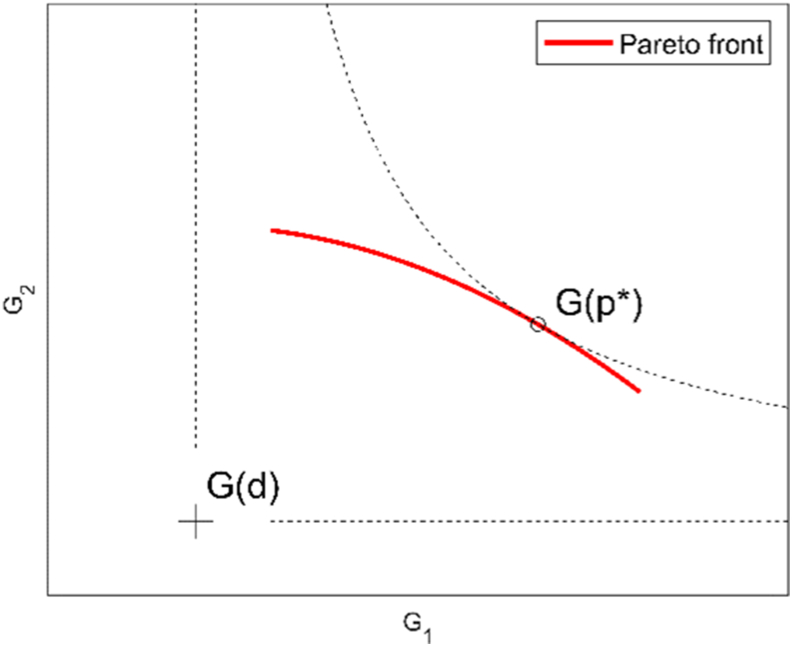
Nash bargaining solution p* identified in the Pareto front of players' gains for a given disagreement outcome d.

From a geometrical perspective, the allocation corresponding to the NBS is identified as the point in which an equilateral hyperbola with origin in the DO (*G1(d)*, *G2(d))* is tangent to the Pareto front (see Fig. 2). An equilateral hyperbola represents the locus of all points having a particular value of the product of the excess payoffs (Eq. (1)). The equilateral hyperbola that maximizes this product for the Pareto optimal strategy profiles must be the one tangent to the Pareto front (the product value would be lower for any other equilateral hyperbola closer to the origin, i.e., intersecting the Pareto front).

It is worth noting that the NBS would “naturally” arise when players fully disclose their strategies and payoffs and are willing to negotiate and cooperate, as it is in the best interest of all players.

### 2.2 The municipal energy system model

Electricity and heating supply in mid-size Swedish municipalities typically consist of a local DH company and one or more (local) electricity companies, which distribute electricity imported from the national grid and generated from local facilities to the users in the municipality [54]. Local electricity generation can comprise combined heat and power plants (CHP) in industry and DH, hydropower, biogas to power units, onshore and offshore wind power, and rooftop or large-scale solar power. DH, CHP in DH, and CHP in the industry mainly use biomass, municipal waste and excess industrial heat. A local electricity supplier can be vertically integrated and manage the local electricity distribution grid as well. Surplus electricity from CHP in DH and industries and from other local generation is utilized within the local scope or is exported to the national grid.

A typical Swedish municipality showing the above setting is Piteå (about 42 000 inhabitants), located in Norrbotten County in the northern part of Sweden. The supplementary material, part A, provides details about the modeled Piteå municipal energy system. The most relevant energy system parameters for this study (year 2015) can be summarized as follows.

- Existing onshore wind capacity of 145 MW and hydropower capacity of 40.9 MW are assumed to be owned by the local utility. These local renewable capacities, with the contribution of 78 MW from industrial CHP and electricity import from the grid, satisfy the annual electricity demand of 1433 GWh.
- The annual heating demand of 211.7 GWh of the consumer group not connected to DH is covered by 24% biomass boilers (50.4 GWh), 28% heat pumps (60 GWh of heat or 24 GWh of electricity considering an annual COP of 2.5), and 48% electric boilers (101.3 GWh) [55,56].
- The total local electricity demand of the as-is situation results in annual CO2 emissions of 56.7 ktCO2 due to the import of grid electricity, considering a Nordic average grid emission factor of 0.156 tCO2/MWh [57].

The electricity and heating sectors of the Piteå energy system were modeled in the advanced simulation tool EnergyPLAN [58]. EnergyPLAN simulates annual energy balances with an hourly resolution and calculates the annual total costs and CO2 emissions of the entire system. An interface was designed between EnergyPLAN and an evolutionary algorithm providing Pareto optimal solutions for a multi-objective optimization problem [59], which was implemented in Matlab. The obtained Pareto fronts (PF) represent the best possible trade-off solutions between the profit functions of the two actors in the municipal energy system. At the same time, the Pareto optimal solutions also represent the candidate game solutions if the players have agreed to cooperate.

The research presented in this paper focuses on two actors (the players of the game): a) a municipal electricity supply company (the “Utility”) and b) a group of consumers representing the heating demand for individual buildings not connected to DH (the “Consumers”). The set of actions available to these two players are.

- Utility: import/export electricity from/to the national grid, investments in different RE technologies, namely onshore wind power (*windON*), offshore wind power (*windOFF*), and large-scale solar PV (*PV*). The Utility sells electricity to the Consumers and charges electricity and grid tariffs. The Utility, due to its small size, is a price taker on the electricity market.
- Consumers: investment among different heating technologies, namely direct electric heating or electric boilers (*EB*), heat pumps (*HP*) and biomass boilers (*BioB*), and a selection of energy efficiency measures (*EE*). The Consumers are a significant electricity user group due to the dominating heating technologies (*EB* and *HP*). These Consumers are therefore assumed to be susceptible to electricity bills according to their choices for heating technology and *EE* measures.

The economic interests of the two actors are represented by their profit or gain functions. For the Utility, the profit function (for one year) is defined as the sum of.

- Annualized costs of the installed RE technologies (based on capital cost, expected lifetime, discount rate, operation and maintenance costs).
- Costs due to electricity import from the grid.
- Revenues from electricity sales to consumers, including grid fees.
- Revenues from electricity export to the grid.

For the Consumers, the profit function comprises costs only.

- Annualized costs of the heating devices (based on capital cost, expected lifetime, discount rate, operation and maintenance costs).
- Annualized costs of *EE* measures in buildings (based on capital cost, expected lifetime, discount rate, operation and maintenance costs).
- Costs for fuel (pellets) for biomass heating.
- Costs for electricity for electric heating, including grid fees.
- Energy taxes and VAT.

Details on the calculations of the two profit functions are given in the supplementary material, part A.

### 2.3 The studied policy instruments

Four policy instruments were studied using the model, aiming to enable higher shares of local RE generation, the switch of heating technologies, and the undertake of *EE* measures, with the higher objective of reducing local electricity grid-related carbon emissions at least by 50%. In a free electricity or energy market, neither a supplier nor a consumer can be forced to act on a carbon target or constraint; hence, this study avoided implementing such a constraint in the model.

In the modeled municipal energy system, carbon emissions solely arise from grid electricity use, and annual carbon emissions were calculated based on a grid emission factor [57]. These emissions can be mitigated by reducing local electricity demand and replacing imported grid electricity with electricity generated from renewable sources within municipal boundaries. Studied policy instruments are carbon pricing (CO2 tax on consumers), financial support for RE technologies (the Swedish electricity certificate scheme and the auction-based Contract for Difference policy), and subsidies for switching to biomass heating and for undertaking *EE* measures in buildings. Table 2**.** presents the values of key model parameters assigned for the Base case (no policy instrument applied) and for the studied policy instruments. The Base case was implemented with ESP = 25 and 40 EUR/MWh, and different discount rates for the Utility were investigated.

**Table 2:** Key parameter values assigned in the Base case and in the studied policy instrument.

Parameter description	Base case value	Investigated values
Annual average electricity spot price: ESP [EUR/MWh]	25 EUR/MWh (considering historical values and futures on NASDAQ as of September 2020)	40 EUR/MWh (high electricity price scenario) International markets increasingly impact Nordic electricity prices.
Biomass (pellets) prices delivered to the doorstep:	7.28 EUR/MWh + 3 EUR/MWh transport costs.	No change in biomass prices in different cases.
Discount rate Utility (*dru*)	9% (representing WACC+1.5%)	3% (a social discount rate) 5% (widely used in ESOM, e.g., in EU-TIMES) 7.5% (representing WACC)
Discount rate Consumers consumer (*drc*)	3%	None.
CO2 tax [EUR/tCO2]	0 EUR/tCO2	30 EUR/tCO2 (recommended by e.g., Ref. [13]) 100 EUR/tCO2 (current Swedish level [60]) 200 and up to 400 EUR/tCO2 (levels as recommended by, e.g., Nordhaus [14])
Electricity certificate scheme	0 EUR/MWh	1.5, 5, 10, 15, 20 and 30 EUR/MWh
Investment grants for *BioB* and *EE measures*	0%	25%; 50%; 75%

Discount rates, as applied in this study, deserve a special mention. The discussion about the “right” discount rate in the cost-benefit analysis of long-term investments is all but over [14,61]. The discount rate can be interpreted as an interest rate for investment decisions, often based on the WACC, or as a social discount rate, which is about the just sharing of costs and benefits between the present and the future. The spectrum of discount rates applied in IAMs and ESOMs spans from low discount rates of 1–2 percent - valuing the future - as in the Stern review [62], to higher discount rates considering the WACC as a minimum to accommodate the need for feasible returns on investments [34,63]. For wealthy households, the implicit discount rate (which reflects preferences, (ir)rational behaviors, and external barriers) may be close to zero for energy investments. In contrast, it may be up to 100% or higher for low-income households [64,65]. This study applied a discount rate of 3% for the investigated Consumers group.

#### 2.3.1 CO2 tax on consumers, protecting industry

This study implemented a CO2 tax (EUR/tCO2) in addition to the existing EU-ETS, which is included in the ESP. The CO2 tax is imposed on private consumers only, protecting the industry (investigated CO2 tax rates as in Table 2). Annual carbon emissions (tCO2/year) from grid electricity import into the scope of the energy model have been calculated in all cases with a National and European Emission Factors for Electricity consumption (NEEFE) of 0.156 tCO2/MWh, which represents an average Nordic value [57]. Biomass use was considered carbon-neutral, and no other fossil fuels were used within the scope of the modeled municipal energy system, which excluded the transport sector.

The total annual CO2 tax amount is calculated from the yearly electricity import in GWh and added as an extra CO2 cost per consumed kWh for all non-industrial electricity users, protecting the heavy industry.

#### 2.3.2 Electricity certificate scheme (ELcert)

The Swedish electricity certificate scheme (ELcert-scheme) was established in 2003, and Norway joined in 2012 [15]. The declared goal is to achieve a new renewable energy generation of 46.4 TWh by 2030 [66]. The ELcert-scheme is a market-based instrument providing a payment - the ELcert-price (EUR/MWh) - for each electricity certificate (1 MWh) generated by a listed RE producer. The ELcert-scheme intended to be technology-neutral and provides a financial incentive for not yet competitive technologies in an otherwise deregulated Nordic electricity market. The stop date for new participants to the scheme was set to December 31, 2021, and the scheme ends in 2035.

A quota system, part of the ELcert-scheme, creates a demand for certificates (quota-obliged electricity use) and a market where these certificates are traded, establishing the ELcert-price. Electricity traders and consumers are obliged by the quota system to buy such certificates and pay to the administrative authority the ELcert-cost, calculated as the ELcert-price multiplied by the quota for a specified year [15].

In the model this was implemented with an ELcert revenue term added to the Utility profit function, limited by the quota-obliged electricity use in the observed geographic scope, considering exemptions for heavy industries. An ELcert-cost term was added to the Consumers’ profit function. This study implemented ELcert-prices as presented in Table 2, considering the historical price development [67].

#### 2.3.3 Contract for difference (CfD)

An auction-based CfD policy instrument is a long-term contract between an electricity generator and the market regulator [16,17]. A CfD finances the gap between the ESP and the Strike Price of new installed RE capacity. The specific implementation of the auction-based CfD can be designed to address the differences in the Levelized Costs of Electricity (LCOEs) of technologies. The authority responsible for the CfD design can consider other characteristics of different technologies, such as annual production profiles, when implementing a specific auction.

This study implemented the LCOEs of the different renewable energy technologies as Strike Prices. These LCOEs vary due to different discount rates, further economic parameters of renewable technologies can be looked up in the supplementary material, part A. The CfD policy in this study is financed through a transfer payment from the Consumers to the Utility. The available CfD budget results from a CO2 tax on the Consumers, implemented in the model as in the CO2 tax policy instrument with rates of 30, 100, 200, and 400 EUR/tCO2. Only this limited CfD budget is available to finance actual Utility investments in new local RE capacities, meaning that financial transfers only occur between market actors within the studied scope and no revenues or costs are generated for the government.

#### 2.3.4 Investment subsidies

Subsidy policy instruments offered by government authorities in the form of financial grants for consumer investments in *EE* measures and *BioB* were considered. Subsidy levels of 25%, 50%, and 75% were implemented, and no budgetary limit was set.

## 3 Results

This section is organized per policy instrument, in the same order as they were introduced in the previous section. First, the results of the two-objective optimization of Utility profit/loss function and Consumers cost function are presented for each policy instrument and set of related parameter values that have been investigated (it is worth recalling that the Pareto optimal solutions represent the candidate solutions of the cooperative version of the game). The figures visualize the Pareto front (PF) between the two objectives, together with other relevant information about the Pareto optimal solutions. The upper part of the figures shows annual CO2 emissions (*CO2tot*) and installed capacities for local renewable energy technologies, namely solar PV (*PV*), onshore wind (*windON*), and offshore wind (*windOFF*). The lower part of the figures shows the overall heating demand (*HTdem*) and the heating supply alternatives, namely heat pumps (*HP*), biomass boilers (*BioB*) and direct electric heating or electric boilers (*EB*).

Then the perspectives of the Utility and the Consumers are analyzed. In this regard, please note that the strategy of the Consumers determines the electricity demand, which impacts the payoff of the Utility (a higher demand would increase sales and profits). On the contrary, without any policy instrument, the actions of the Utility do not affect the costs to the Consumers (because the local Utility is a price taker on the Nordic electricity market and has no influence on the ESP, which is the base component for the electricity cost paid by the Consumers). The analysis of the perspectives of the players leads to identifying the Nash Equilibrium (NE) of the non-cooperative version of the game, as well as the disagreement outcome (DO) and Nash Bargaining Solution (NBS) of the cooperative version of the game. Finally, the impacts on emission reduction and the costs or revenues for the government are determined. Further details and results, in addition to those presented in this section, can be found in the supplementary material, part B.

### 3.1 Base case – no policy instrument

**Fig. 3:**
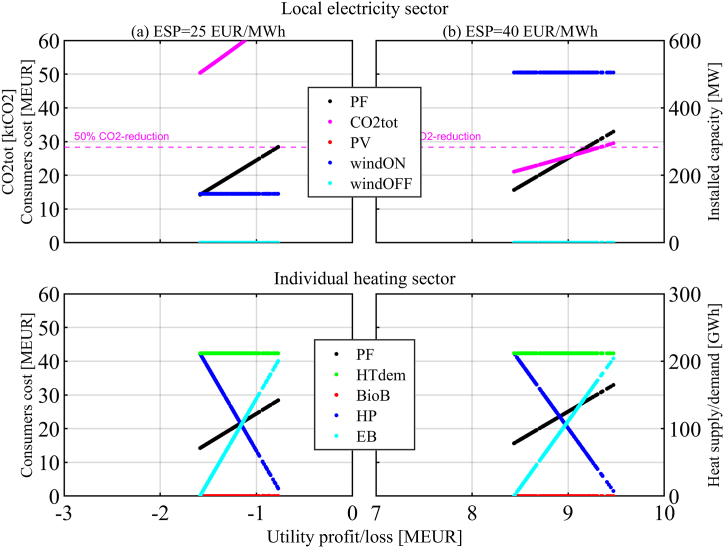
Base case – no policy instrument. Pareto optimal sets and front (PF), CO2 emissions, installed capacities in the local electricity sector, and heat supply in the individual heating sector. Discount rates: dru = 9%, drc = 3%.

The result for the Base case without policy instruments is shown in Fig. 3. Fig. 3 (a)-column presents the results for an ESP of 25 EUR/MWh, while Fig. 3 (b)-column covers an ESP of 40 EUR/MWh.

A generic point on the PF of the Base case represents a Pareto optimal solution where the Utility either does not invest at all (Base case with ESP = 25 EUR/MWh) or invests in the maximum allowed *RE* capacity (Base case with ESP = 40 EUR/MWh), and where the Consumers choices represent a mix between *HP* and *EB*. A generic point on the PF is not a NE because Consumers can find a better strategy by using more *HP,* resulting in reduced Utility profits.

Not investing is the best strategy for the Utility with an ESP of 25 EUR/MWh, regardless of what the Consumers do. For the Consumers, investments in *BioB* and *EE* measures are not convenient under any Base case conditions, and the annual heating demand remains at 211.7 GWh (*HTdem* in Fig. 3). Investing in 100% *HP* reduces Consumers' costs by 35% (from 22 MEUR to 14.2 MEUR) and lowers electricity demand for heating by 32% (from 125.3 GWh to 84.7 GWh) compared to the as-is situation. The Utility has no strategy available to counter the effect of reduced sales. Table 3 presents a simplified version of the generalized form of the strategic game, where the payoffs of the two players (Utility and Consumers) are represented with plus and minus signs. The only Nash equilibrium (NE), in which each player's gain is maximum regardless of the strategies chosen by the other player, is found when the Consumers cannot use more *HP* and the Utility does not invest (cell with **U+ C+** in Table 3). In this case, the NE, which is the disagreement outcome (DO) in the cooperative game, is also the Nash bargaining solution (NBS) because it is on the PF (i.e. there are no excess payoffs).

**Table 3:** Payoffs for the Base case – no policy instrument with ESP = 25 EUR/MWh.

		Consumers	
		HP	EB
Utility	Invest	U– C+	U– C–
	No invest.	**U+ C+**	U+ C–

Emissions are reduced by 11% due to the lower electricity use for covering the heating demand, not achieving the 50% mitigation target (*CO2tot* in Fig. 3).

For the Base case with an ESP of 40 EUR/MWh, cet. par. (in Fig. 3 (b)-column), the best strategy for the Utility becomes investing in RE regardless of what the Consumers do. Investments in *windON* are economically feasible for all *dru*, PV becomes a viable investment with an ESP of 40 EUR/MWh when considering a lower *dru* of 5%. Investing in *windOFF* is not convenient, even with the higher ESP and a low *dru*. The only NE (also DO and NBS) is found when the Consumers cannot use more *HP* and when the Utility invests in the maximum capacity of the economically feasible RE (Table 4). Emissions are mitigated to and below the 50% target due to the reduced import of grid electricity. Finally, the Base case without policy instruments has no cost or revenue for the government or the general taxpayer.

**Table 4:** Payoffs for the Base case – no policy instrument with ESP = 40 EUR/MWh.

		Consumers	
		HP	EB
Utility	Invest	**U+ C+**	U+ C–
	No invest.	U– C+	U+ C–

### 3.2 CO2 tax on consumers, protecting industry

**Fig. 4:**
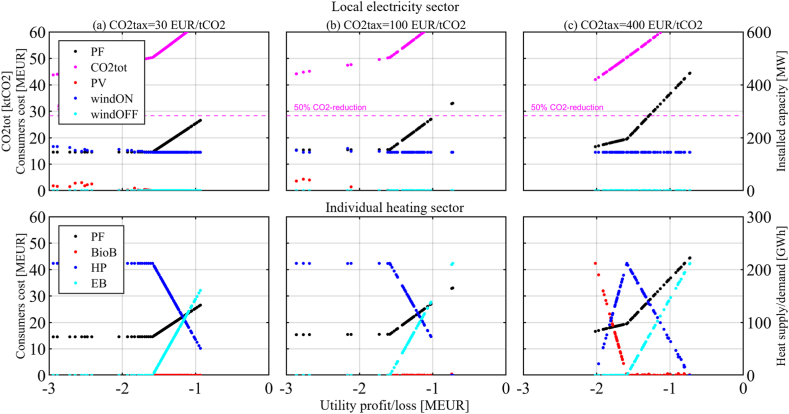
CO2 tax on consumers, protecting industry. ESP = 25 EUR/MWh. CO2 tax rates = 30, 100, and 400 EUR/tCO2.

The results in Fig. 4 (a) and Fig. 4 (b) show that Consumers will do their best to lower the additional CO2 tax costs by investing in *HP*. If they use 100% *HP*, a CO2 tax of 100 EUR/tCO2 results in 1.4 MEUR additional costs for the Consumers, and a CO2 tax of 400 EUR/tCO2 in 5.7 MEUR (Fig. 4 (c)). However, in the case of the high CO2 tax rate of 400 EUR/tCO2, it becomes convenient for the Consumers to switch to *BioB* (Fig. 4 (c)-column), while the Utility has no incentive to invest in general. Utility investments in RE would reduce the import of electricity, mitigate emissions and help the Consumers reduce costs, but they also dramatically increase Utility losses because an ESP of 25 EUR/MWh is too low; hence these solutions, although Pareto optimal, can be disregarded in the analysis of the strategies available to the players and are cut from the diagrams. *EE* measures are not economically feasible for the studied CO2 tax rates, keeping the annual heating demand constant at 211.7 GWh.

The NE is again found when Consumers use 100% *HP*, unless the CO2 tax rate gets very high and Consumers choose 100% *BioB* in the NE. Emissions are mitigated by 11% with investments in 100% *HP*, and by 26% with investments in 100% *BioB*. The government accrues a CO2 tax revenue of 5.0 MEUR with a CO2 tax rate of 100 EUR/tCO2, and 20.2 MEUR with a CO2 tax rate of 400 EUR/tCO2 when Consumers use 100% *HP*. When switching to BioB at a CO2 tax rate of 400 EUR/tCO2, the Consumers would not contribute to the expected CO2 tax revenue of 16.8 MEUR.

### 3.3 Electricity certificate scheme (ELcert-scheme)

**Fig. 5:**
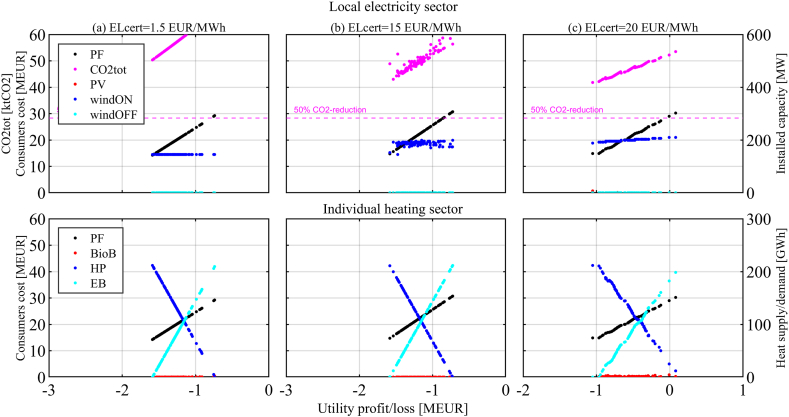
Electricity certificate scheme. ESP = 25 EUR/MWh. ELcert-prices = 1.5, 15, and 20 EUR/MWh.

The ELcert-scheme provides additional revenue to the Utility for new RE capacities. Fig. 5 presents the results for three different ELcert-prices (1.5, 15, and 20 EUR/MWh) and an ESP of 25 EUR/MWh.

This study assumes that already existing local wind capacity has been financed differently and is therefore excluded from the ELcert-scheme. With these assumptions, no new investments are economically feasible for the Utility with ELcert-prices far below 15 EUR/MWh (Fig. 5 (a)-column). The ELcert-price of 15 EUR/MWh (Fig. 5 (b)-column) shows that the Utility invests the available ELcert-budget in new RE (*windON*). ELcert-prices of above 15 EUR/MWh justify investments but are limited by the quota-obliged electricity use (all non-industrial use) within the scope of the modeled municipality (for example, with ELcert-price of 20 EUR/MWh in Fig. 5 (c)-column). The quota system limits installed capacities, and while additional profits grow with increasing ELcert-price (Fig. 5 (c)-column) an increased quota would of course increase capacities. The additional costs for the Consumers due to the ELcert-scheme are minor and do not change the strategies chosen by the Consumers compared to the Base case.

From the point of view of game theory, the situation observed for the ELcert-scheme is similar to the Base case. For ELcert-prices below 15 EUR/MWh, the only NE (also DO and NBS) is found when the Consumers cannot use more *HP* and the Utility does not invest. For ELcert-prices that justify Utility investments in RE (Fig. 5 (b) and (c)-columns), the NE is found when the Utility invests in RE capacity while the Consumers cannot use more *HP*. CO2 emissions are mitigated by 26% with an ELcert-price of 20 EUR/MWh (Fig. 5 (c)-column). The ELcert-scheme neither creates costs nor revenues for the government.

### 3.4 Contract for difference (CfD)

**Fig. 6:**
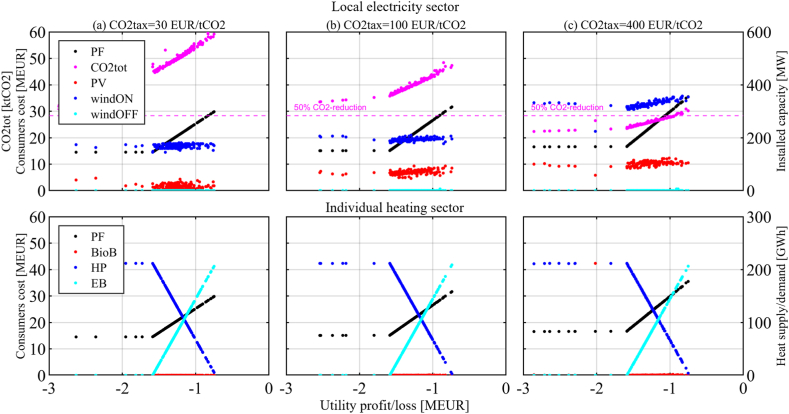
Contract for Difference with different CO2 tax rates. CO2 tax rate = 30, 100 and 400 EUR/tCO2.

Fig. 6 presents the results for the CfD policy instrument with CO2 tax rates of 30, 100, and 400 EUR/tCO2 in Fig. 6 (a), (b), and (c)-columns, the ESP being 25 EUR/MWh and discount rates *dru* = 9% and *drc* = 3%. With higher CO2 tax rates, and hence with a growing CfD-budget available, it can be observed that investments in new RE capacities become increasingly convenient to the Utility. As the Utility invests and the Consumers switch to *HP*, less electricity needs to be imported, resulting in a reduced CO2 tax budget, not allowing the Utility to increase investments.

None of the PF solutions is a NE, because both Utility and Consumers can find better strategies than their PF strategy while the other actor keeps the own. The NE for the CfD policy instrument, which is also the disagreement outcome (DO), is found when Consumers reduce as much as they can their electricity consumption in an attempt at lowering their costs. At the same time, the Utility makes no investments to keep its losses as low as possible, as both actors are trying not to give the other any advantage.

**Fig. 7:**
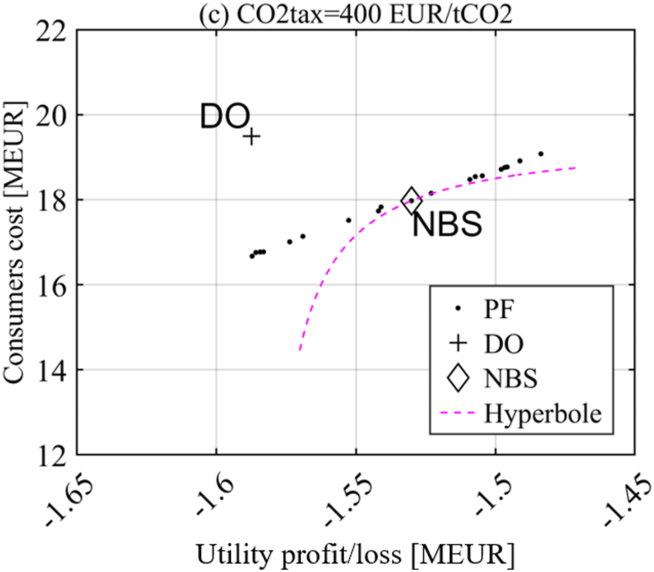
CfD - Disagreement outcome (DO) and Nash bargaining solution (NBS) CO2tax = 400 EUR/tCO2.

Among the investigated policy instruments, only the CfD presents a Nash Bargaining Solution (NBS) different from the NE/DO. However, the NBS is very close to the NE/DO as shown in Fig. 7, which zooms closely in on the PF in Fig. 6 (c)-column.

Emissions are both mitigated by the strategy of the Consumers (almost 100% *HP*) and by the Utility investments in RE that utilize the available CfD budget from the CO2 tax on the Consumers. Mitigations reach 16% (a), 36% (b), and 56% (c) in Fig. 6 for the respective NBSs. This policy instrument – a CfD for the local Utility financed by transfers from a CO2 tax on local Consumers – has low additional costs to the Consumers and no additional cost to the government.

### 3.5 Investment subsidies

**Fig. 8:**
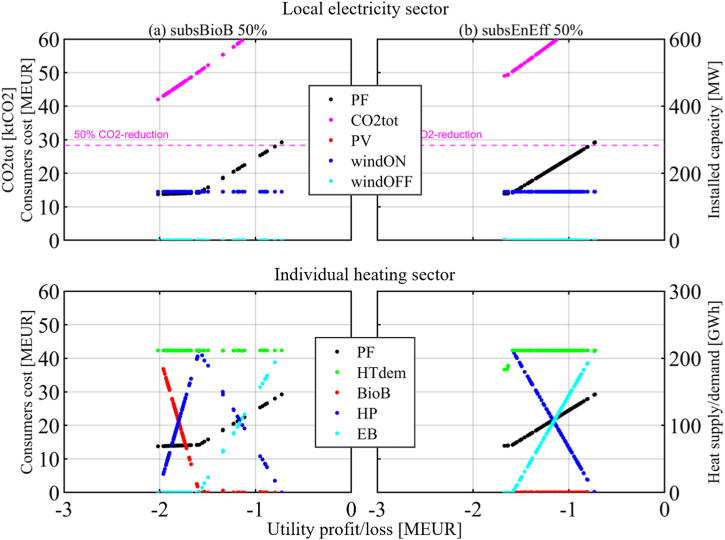
Investment subsidies. ESP = 25 EUR/MWh. (a) 50% subsidy for biomass boilers; (b) 50% subsidy for energy efficiency measures.

Investment subsidies of 25%, 50%, and 75% have been investigated for *BioB* and *EE* measures without setting a budgetary limit. Other parameters are as in the Base case. Fig. 8 presents the results for a 50% subsidy level and an ESP of 25 EUR/MWh (Fig. 8 (a)-column for a BioB subsidy level of 50% and Fig. 8 (b)-column for the respective EE subsidy level).

*BioB* become the most convenient choice for Consumers only when they are subsidized at about and above 50%. Similar subsidy levels encourage Consumers to invest in *EE* measures, reducing electricity demand by about 15%. The Utility experiences a loss of sales as electricity demand is reduced, and has no strategic action available to counteract the situation. Investment in *HP* combined with subsidized *EE* measures results in fewer Utility losses than in the *BioB* subsidy case.

Regardless of the subsidy level provided to the Consumers, the NE (also DO and NBS) is the leftmost solution on the PF. The Utility does not invest, and the Consumers do whatever is more convenient to reduce their costs considering the available subsidies.

Subsidy policy instruments do not allow the targeted 50% mitigation of emissions to be reached in the studied context. The costs to the government are 19.7 MEUR for *EE* measures and 58.7 MEUR for *BioB* (about 2000 EUR and 6000 EUR, respectively, per household).

## 4 Discussion

This section discusses the results for the Base case and the four policy instrument cases of CO2 tax, electricity certificate scheme (ELcert), Contract for Difference (CfD), and investment subsidies for energy efficiency measures and biomass boilers.

**Fig. 9:**
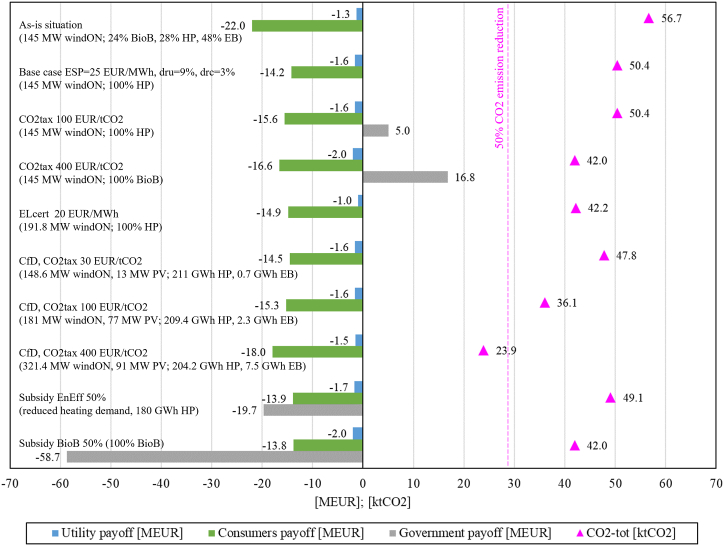
Results (NBS) from selected policy instruments. All with an ESP = 25 EUR/MWh. Utility, Consumers and Government payoffs [MEUR]. CO2-tot emissions [ktCO2].

Fig. 9 compares the NBSs of the studied policy instruments in relation to the as-is situation – represented at the top of Fig. 9 – where the investigated Consumers group covers its annual heating demand of 211.7 GWh with a heating technology mix of 24% *BioB*, 28% *HP* and 48% *EB* for a total cost of 22 MEUR. The Utility, which owns and operates an onshore wind capacity of 145 MW, accrues losses of 1.3 MEUR in the as-is situation.

The best strategy available to the Consumers for all studied policy instrument cases is either to use 100% *HP* or 100% *BioB*, combined with energy efficiency measures when subsidies are sufficiently high. This strategy results in lower heating costs, lower electricity demand, and subsequently in lower Utility sales and profits for all situations in which the Utility has no economically viable strategy to counter the choice of the Consumers. Emissions drop by 11% (100% *HP*), by 13% (100% *HP* and *EE*), and by 26% (100% *BioB*) as less electricity needs to be imported from the grid, while the Utility does not invest in new *RE*. In the following the four specific policy instruments are discussed one at a time.

As Consumers already choose *HP* without any policy instrument, as shown in the Base case, the CO2 tax appears to be ineffective for mitigating emissions from this specific heating sector in the studied context, unless the tax rate is set far beyond recommended levels of 30 EUR/tCO2 [13] and the current Swedish level of about 100 EUR/tCO2. A CO2 tax provides revenue to the government, but an unreasonably high CO2 tax rate (400 EUR/tCO2) would need to be introduced for achieving higher emission reductions by investing in *BioB* or *EE*.

The following presented case in Fig. 9 represents the existing Swedish policy instrument of the electricity certificates scheme (ELcert-scheme) with an ELcert-price of 20 EUR/MWh, which reflects historic levels of certificate prices [15,67]. The certificate market dynamically determines the actual ELcert-price, depending on installed renewable capacity and the legally regulated demand for certificates (quota system). A Utility investing in RE would accrue profits or losses depending on the sum of the prevailing electricity price and the certificate price. Since the introduction of the ELcert-scheme in 2003, both periods with over- and undersupply of renewable capacity and certificates have been observed [68], the profits of the investors being affected by under- or overcompensation. Despite uncertainties about the profitability, the scheme successfully incentivized significant investments in RE capacities in Sweden and Norway, including wind energy, biomass in CHP, and hydropower [15,69]. This was achieved without imposing unreasonably high costs on electricity consumers, hence efforts to reduce electricity consumption in the heating sector by energy efficiency measures as a direct result of the ELcert-scheme could not be observed. By 2022, about one million households in Sweden still use direct electric heating as the primary heating technology, even though often in combination with low-cost air-to-air HP or different forms of biomass heating [56]. This study confirms that with increasing ELcert-prices the best strategy for the Utility is to invest in RE as soon as the accrued revenue from the ESP and the ELcert-price exceeds the LCOE of a technology. The best strategy for the Consumers does not change compared to the Base case because the ELcert-cost is insufficient to affect their choices, being merely a component of the actual electricity cost paid by the Consumers (which is the sum of the ESP, the certificate cost, grid tariffs, energy taxes, and 25% VAT [70]). Mitigating electricity production-related emissions with the market-based ELcert-scheme does not create additional costs (or revenues) for the parts of the society outside the scope of the electricity system. It causes only minor cost increases to electricity consumers, even when the ELcert-price is relatively high.

Contrary to the ELcert-scheme, CfD policies provide long-term certainty to investors by financing the gap between ESP and LCOE and avoiding under- or overcompensation. The Utility investments are compensated through the CfD, and the Consumers still experience a financial advantage (compared to the as-is situation) by switching to *HP*, although reduced by the CO2 tax that is part of CfD design implemented in this study. Of course, relatively high CO2 tax rates of above 200 EUR/tCO2 are needed to reach emission reduction levels close to 50% with this policy instrument as well. This direct transfer instrument enables an interaction between supplier and consumers, since the CO2 tax increases the costs for electricity use and the transfer payment incentivizes investments in RE that in turn reduce the burden of the CO2 tax. Contrary to the case of the CO2 tax policy instrument, the Consumers group does not choose biomass boilers with higher CO2 tax rates. This is due to the Utility investments into RE, which reduce electricity imports and related emissions, resulting in a lower overall tax burden for the Consumers. The CfD policy instrument does not generate a revenue or a cost to the government. Still, the policymaker is challenged to carefully adjust the CO2 tax levels over time to achieve the envisioned emission targets and to guarantee transfer payments during the contracted CfD period. The investigated design of the CfD with a local transfer payment from the specified heating customers group (and other electricity users) suggests that such a policy instrument could be practical and effective within a local context, where a locally-based Utility could offer such a scheme to its local customers.

Finally, the two subsidy cases are shown in Fig. 9. *EE* measures and *BioB* are capital intensive and require high subsidies to become economically convenient to the considered Consumers group in the Nordic context. A policymaker should be aware that the other investigated policy instruments have little effect on the best strategy of the Consumers and, therefore, should not disregard the costly subsidies. Such subsidies can enable households to participate in the transition process by investing in non-electric heating technologies and energy efficiency retrofits. These investments, in addition to reducing electric energy consumption (MWh), would also comprise other benefits, including the reduction of electricity peak demand (MW), the improvement of building stock and a local economic stimulation, since subsidies have the potential of being complemented with private investments [71,72]. An effective design of such subsidies should consider the household financial status, age, standard, and location of the building [73].

Policy instruments should enable Utility investments into local RE and ensure long-term certainty for them while avoiding overcompensation. Consumers would invest in *HP* even without subsidies, while other technologies or measures only become convenient with policy instruments such as high CO2 taxes or subsidies. Investment subsidies are costly to the government, but have the potential to unlock private financing and create additional value. Required levels of effective financial incentives and CO2 tax rates depend on several parameters, among which technological development (resulting in lower LCOEs), levels of electricity prices (ESP), applied discount rates, household incomes, and of course on the structure of the investigated electricity system with the associated grid emission factor.

## 5 Conclusion and policy implications

The applied game theory-based approach provides insights on policy instruments as the two selected actors in a Nordic municipality (a local electricity supplier and a heating consumer group not connected to district heating) react to their implementation with their own optimal strategies. It was surprising to observe that the level of strategic interaction between these actors is not enough to create through cooperation a common surplus to be fairly allocated between them. In fact, under current market conditions the cooperative Nash Bargaining Solution is equal or very close to the non-cooperative Nash Equilibrium for most of the investigated policy instruments.

The most suitable instrument to achieve relevant CO2 emission reductions in the modeled municipal energy system is the auction-based Contract for Difference policy. This has the potential to be designed for and applied in a local context and actually enables a higher level of interaction between the two considered actors. In fact, the local Utility would receive financing for a renewable investment program through a transfer payment (e.g., a CO2 tax or fee) from its local customers, with modest additional costs to them and no costs to the government. The main challenges of such a policy are to set the right level of transfer payment, to adjust it over time, and to guarantee the financial support over the contracted period.

As for the other considered policy instruments.

- The CO2 tax, a policy instrument that increases the electricity cost paid by the users, would need to set high tax rates in the observed Nordic context, characterized by low electricity prices and a low grid emission factor, to incentivize heating technology options other than heat pumps (which are already the best option under no-policy circumstances).
- The market-based Swedish electricity certificate scheme, introduced in 2003 and financed by all electricity consumers (except energy-intensive industries), incentivizes renewable energy generation from wind, solar photovoltaic, hydropower, and biomass combined heat and power, but is affected by some level of uncertainty about long-term investments and has little impact on the heating technologies chosen by the consumers. Like the CO2 tax, this policy instrument also increases the electricity cost for the user, but the increment is relatively low and there are no costs or benefits for the government.
- Subsidies for investments in, e.g., energy efficiency measures or replacing electric heating with biomass boilers, should not be disregarded despite the high cost for the government. In fact, they can stimulate private initiatives and unlock private financing, contributing to the local economic development.

In order to significantly mitigate emissions from electricity generation, policy instruments targeting suppliers are required to close the gap between the electricity system price and the levelized cost of energy, to avoid under- and overcompensation of investors, and to guarantee long-term economic certainty. Ideally, all consumer groups should contribute to financing such policies. On the other hand, achieving emission mitigation contributions from the consumer side with those policy instruments appears to be difficult, as, e.g., very high CO2 tax rates would have to be introduced. Despite the costs to the government, investment subsidies for non-electricity heating technologies and energy efficiency retrofits of buildings should be considered as complementing policies.

The results obtained in this paper are also relevant outside the Nordic electricity and heating market as they can be transposed to markets with higher electricity prices and energy systems with higher grid emission factors, where effective tax rates or subsidies would be lower compared to the Nordic context. Heat pumps appear to be economically feasible for households not connected to district heating under almost any market conditions, even without financial incentives provided by policy instruments.

The limitations of the game theory-based approach seem to be mostly related to the number of actors that can be considered at once in the definition of the energy system optimization problem. However, when the number of actors increases, a possible countermeasure to reduce the computational complexity is to consider only a discrete number of available strategies for each actor rather than using continuous decision variables to describe them.

In some future work it would be interesting to further extend the analysis of the policy instruments within a municipal energy system with this game theory-based approach by including new actors that may have strategic interactions with the two already considered in the present paper. The scope of the model could be expanded to include the transport sector, which has a lot of potential intersections with the electricity and heating sectors, so that a group of consumers may choose among biofuels, hydrogen and electricity to power their vehicles. Another possible direction to expand the work could be the inclusion of other technologies (e.g., carbon capture, utilization and storage, energy storage technologies like batteries) among the investment options available to the actors.

## Data availability statement

Inquiries about the dataset supporting the findings of this study can be directed to the corresponding author.

## CRediT authorship contribution statement

**Robert Fischer:** Writing – original draft, Software, Methodology, Investigation, Formal analysis, Data curation, Conceptualization. **Andrea Toffolo:** Writing – review & editing, Validation, Supervision, Software, Methodology, Formal analysis.

## Declaration of competing interest

The authors declare that they have no known competing financial interests or personal relationships that could have appeared to influence the work reported in this paper.

## Nomenclature

BioB: Biomass boilers
CfD: Contract for Difference
CGE: Computable general equilibrium
CHP: Combined heat and power plant
CO2: Carbon dioxide (typically with the meaning of CO2 equivalent in the context of the manuscript)
CO2tot: annual CO2 emissions within the scope of the investigated model
COP: Coefficient of performance (an expression of the efficiency of a heat pump)
DICE: dynamic integrated climate-economy model
DH: District heating
DO: Disagreement outcome
dr: Discount rate
drc: Discount rate for the consumers
dru: Discount rate for the utility
EB: Electric boiler
EE: Energy efficiency
ELcert: Electricity certificate scheme (established in Sweden and Norway)
EnergyPLAN: A deterministic model which simulates the operation of a given energy system
ESM: Energy system modeling
ESOM: Energy system optimization and modeling
ESP: Electricity spot price
ETS: Emission Trading Scheme or Emissions Trading System
EUR: Euro
GHG: Greenhouse gases
GT: Game theory
HP: Heat pump
HTdem: heating demand of the investigated sub-sector
IAM: Integrated assessment model
IEA: International Energy Agency
IPCC: Intergovernmental Panel on Climate Change
ktCO2: kilotonnes of CO2
LCOE: Levelized costs of electricity
MARKAL: MARKAL is a numerical model used to carry out economic analysis of different energy related systems at the country level to represent its evolution over a period of usually of 40–50 years. The word MARKAL was generated by concatenating two words (MARKet and ALlocation)
MILP: Mixed integer linear programming
MOEA: Multi objective obtimisation with evolutionary algorithms
MOOP: Multi objective optimization problem
NBS: Nash bargaining solution
NE: Nash equilibrium
OSeMOSYS: Open Source energy MOdelling SYStem
PF: Pareto front
PRIMES: PRIMES (Price-induced market equilibrium system), energy system model designed to project the energy demand, supply, prices, trade and emissions for European countries and assess policy impacts
PV: Photovoltaics
NASDAQ: National Association of Securities Dealers Automated Quotations
NEEFE: National and European Emission Factors for Electricity consumption
RD&D: Research, development and demonstration
RE: Renewable Energy
tCO2: tonnes of CO2 (typically with the meaning of CO2 equivalent)
TIMES: TIMES is a technology rich, bottom-up model generator, which uses linear-programming to produce a least-cost energy system, optimized according to a number of user constraints, over medium to long-term time horizons
WACC: Weighted average cost of capital
windOFF: offshore wind power
windON: onshore wind power
WITCH: World Induced Technical Change Hybrid Model
