# Supplementary material for: Game theory-based analysis of policy instrument consequences on energy system actors in a Nordic municipality
Source: Heliyon. 2024 Feb 4;10(4):e25822. doi: 10.1016/j.heliyon.2024.e25822 (PMC10881334; doi:10.1016/j.heliyon.2024.e25822)
Supplement: Multimedia component 1 [file mmc1.docx]

**Game theory-based analysis of policy consequences on energy system actors in a Nordic municipality**

Robert Fischer^*,a^, Andrea Toffolo^a^

^a^ Energy Engineering, Division of Energy Science, Luleå University of Technology, SE-97187 Luleå.

^*^ Corresponding author: [robert.fischer@associated.ltu.se](mailto:robert.fischer@associated.ltu.se)

**Supplementary material, Part A**

**The Piteå case study in EnergyPLAN**

Piteå municipality, located in Norrbotten county of Sweden, is a representative municipality in the Nordic context. Piteå hosts large-scale forestry, pulp, and paper industries, which dominate the economic sectors. Urban areas are heated by district heating, fuelled by industrial excess heat and biofuels, and areas not connected to district heating mainly use electricity and biofuels for individual heating. Industries and heating characterize electricity consumption. A case study focusing on the electricity and the individual heating sector of the Piteå municipal energy system was implemented in EnergyPLAN as part of the INTERREG project Arctic Energy between 2016 and 2018 [1]. Based on this case study, optimal solutions for integrating electricity and individual heating sectors have been investigated in [2].

Key figures about Piteå’s electricity and heating sectors in 2015 are presented in Table 1. Table 1 also shows projections for the years 2020 and 2030, which are based on expected population and GDP growths and assumed efficiency gain [3–8].

Table 1: Piteå municipality, key figures 2015, projections for 2020 and 2030

| ***Piteå municipality, key figures 2015*** | ***Unit*** | ***2015*** | ***2020*** | ***2030*** |
| --- | --- | --- | --- | --- |
| Population^a, c^ |  | 41 548 | 42 055 | 43 069 |
| Piteå (administrative seat)^b, c^ |  | 23 067 | 23 405 | 24 081 |
| Land area^d^ | km^2^ | 3 086 | 3 086 | 3 086 |
| Housing - number of dwellings^e^ |  | 19 273 | 20 100 | 21 753 |
| In residential buildings |  | 11 509 | 11 726 | 12 159 |
| In apartment buildings |  | 7 384 | 7 874 | 8 854 |
| In other buildings |  | 380 | 440 | 740 |
| Total final energy consumption^f^ | GWh | 5 901 | N/A | N/A |
| Total final electricity consumption^g^ | GWh | 1 453 | 1 433 | 1645 |
| Municipal electricity production^h^ | GWh | 1 117 | 1437 | 2497 |
| Industrial CHP^i^ | MW \| GWh | 78 \| 499 | 78 \| 499 | 78 \| 499 |
| Hydropower^j^ | MW \| GWh | 40.9 \| 221 | 40.9 \| 221 | 40.9 \| 221 |
| Windpower^k^ | MW \| GWh | 145 \| 397 | 248.2 \| 712 | 625 \| 1766 |
| Solar PV^l^ | MW \| GWh | 0.26 \| 0.20 | 5 \| 5.4 | 10 \| 11 |
| Total heat production by DH^m^ | GWh | 269 | 269 | 242 |
| Industrial waste heat^i^ | GWh | 257 | 257 | 232 |
| Heating centers (boilers)^n^ | GWh | 11 | 11 | 10 |
| Total heat energy consumption (no DH)^o^ | GWh | 238 | 211 | 190 |
| Electricity (direct) | GWh | 146 | 101 | 60 |
| Heat pump | GWh | 20 | 60 | 100 |
| Biomass | GWh | 70 | 50 | 30 |
| Oil | GWh | 2 | 0 | 0 |

**a, b** [9]*-BE0101; **c** [7]; **d** [9]-MI0802AA; **e** [9]-BO0104AE, BO0104AG; **f** [9]-EN0203AE total final energy consumption is not within the scope of this study; **g** [9]-EN0203AE, [10] 2020 estimates consider efficiency measures as proposed in Piteå SEAP, for 2030 it is assumed that electricity consumption increase follows national estimations [3–5]; **h** [9]-EN0203AD, [11]; **i** [12], no growth estimates for energy generation in paper and pulp industries are available; **j** [9]-EN0203AD, [13], environmental legislation does not permit expansion of hydropower [14]; **k** [9]-EN0203AD; **l** [13]; **m** [9]-EN0203AC, [10] for 2020 it is assumed that district heating production increases due to population growth and housing growth is balanced by energy efficiency measures, for 2030 additional 10% heating demand reductions are assumed; **n** [11], 2020 and 2030 values remain about the same as heating centers are only used during industrial outages; **o** [9]-EN0203AE, [11], [15,16], estimates for 2020 are based on proposed SEAP measures, for 2030 additional 10% heating demand reductions are assumed, electric heating and biomass continue to reduce and heatpumps increase;

*) references to SCB [9] include the reference code to the specific data.

**Elements for the calculations of total system cost function and profit functions for utility and consumers**

***Discount rate dr***

Discounting in energy system analysis considers two perspectives: social *dr*, for evaluating costs and benefits from a societal perspective, and individual *dr*, for evaluating investment decisions [17]. Applied social *dr* in energy studies ranges between 1% and 7%. The *dr* for industrial investors ranges from 6% to 15% [17,18] and must be equal to or higher than the weighted average cost of capital (WACC). For wealthy households, the implicit *dr* may be close to zero for energy investments, whereas for low-income households, it may be up to 100% and higher [19,20]. For this study, a *dr* of 9% was applied in calculating the total system cost function in EnergyPLAN and the profit function for the Utility (*dru*); for the investigated Consumers group, a *dr* of 3% (*drc)* was applied.

***Technology costs***

Table 2 presents the Levelized Costs of Electricity *LCOE* in EUR/MWh for the considered renewable electricity generation technologies and the annualized costs *C_ann_* for electric heating, biomass boilers, and a 50%/50% mix of ground source and air-air heat pumps. A *dru* of 9% is used in calculating *LCOEs* and a *drc* of 3% for *C_ann_* for the heating costs. *LCOEs* and *C_ann_* take into account the technology cost projections for 2030 and are based on average capacity factors for the Nordic location. Technology cost parameters are taken from the EnergyPLAN package [21]. References of the EnergyPLAN cost database include the catalogs of energy technology data published by the Danish Energy Agency and Energinet, JRC Technical Reports, and the ETRI projections for 2010–2050 [22–24].

Table 2: LCOE for Solar PV, onshore and offshore wind (discount rate dru of 9%); C_ann_ for electric heating, biomass boilers, and a 50%/50% mix of ground source and air-air heat pumps (calculations based on 2030 technology cost parameters and a discount rate drc of 3%).

|  |  | ***LCOE* [EUR/MWh]** | | |
| --- | --- | --- | --- | --- |
| **Renewable Energy Technology** | **Capacity Factor** |  | ***dru* = 9%** |  |
| Solar PV | 12% |  | 50 |  |
| Wind onshore | 30% |  | 40 |  |
| Wind offshore | 40% |  | 68 |  |
|  |  |  | ***C_ann_* [EUR/unit]** |  |
| **Heating Technology** |  |  | ***drc* = 3%** |  |
| Electric heating |  |  | 236 |  |
| Biomass boiler |  |  | 1158 |  |
| Heat pump mix |  |  | 652 |  |

***Energy efficiency measures***

Costs of energy efficiency measures resulting in energy savings in the individual heating sector have been compiled for this paper from several references relevant to the studied building sector [25–30]. The relationship between investment costs for a single-family home and potential energy savings is illustrated in Figure 1.


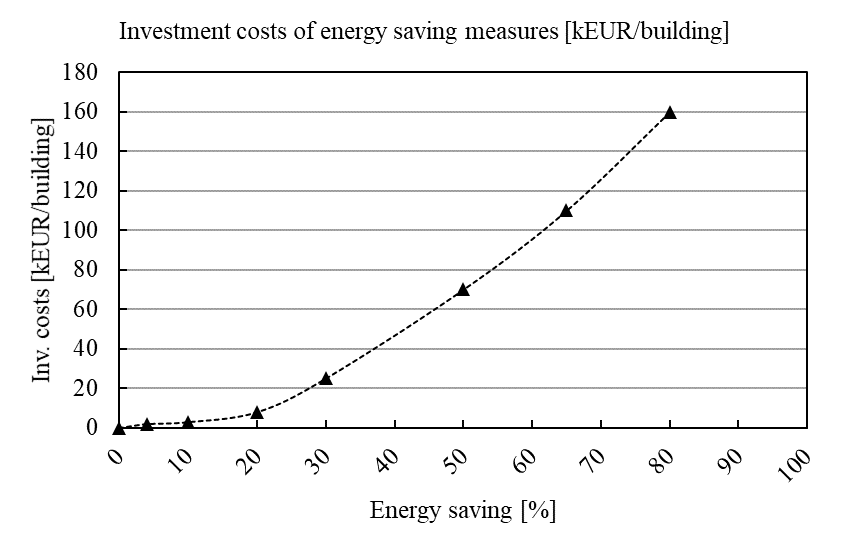


Figure 1: Investment costs for energy efficiency measures in a single-family home.

***Electricity spot price ESP***

Calculated costs for imported electricity and revenues for sold electricity are based on the hourly electricity spot price ESP for the Nordic electricity system, as available from the NordPool power market [31]. The selection of the ESP value is based on the historical price trend of the Nordic power market, where the average annual electricity spot price moved between a long-term low of 21 EUR/MWh in 2015 and 45 EUR/MWh in 2018 (Figure 2, [32]). The values considered in the paper for the annual average ESP are 25 and 40 EUR/MWh.


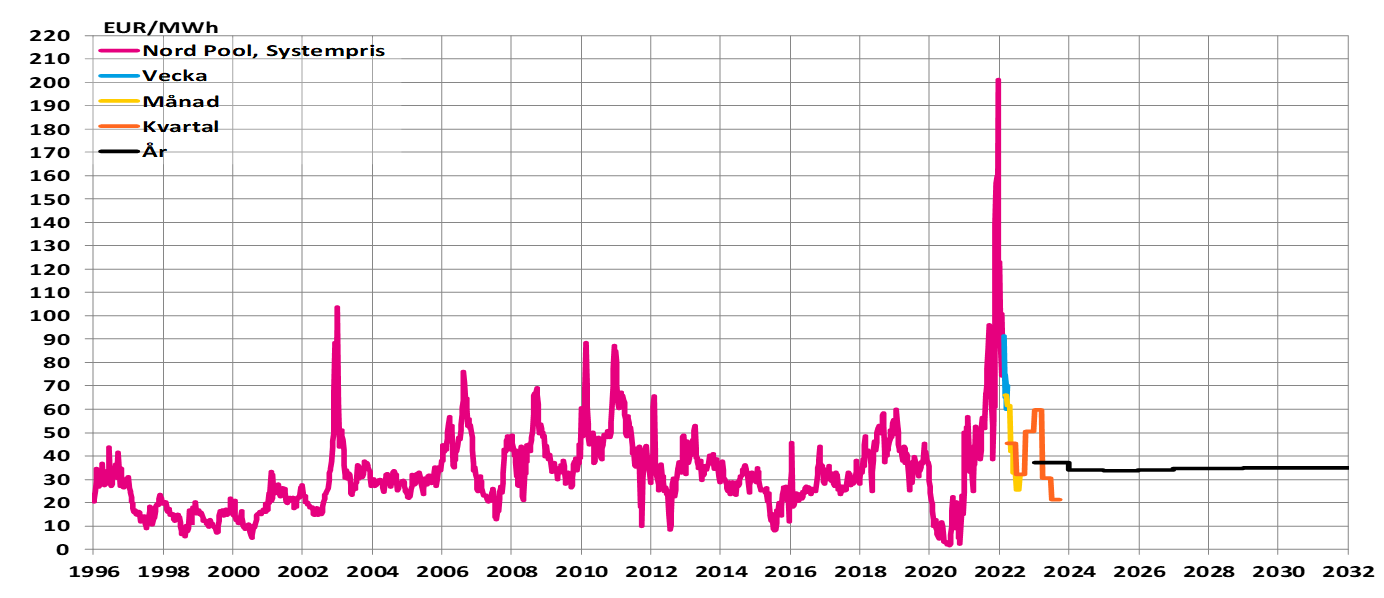


Figure 2: Electricity spot prices Nordpool (system price)

***Grid fees, energy tax, and VAT***

The utility profit function in this study considers revenues from grid fees. The distribution grids in Sweden are owned and operated by about 170 different companies. Grid fees and utility profits from grid provision are regulated by the authority Energimarknadsinspektionen in Sweden [33]. This authority classifies consumers into 15 groups, so grid fees differ widely between small electricity consumers and industry. Grid fees can be set as annual average fees or consider high and low load periods. Grid fees also depend on the connection size of the consumer (limiting the maximum electricity demand). This study considers an annual average grid fee of 40 EUR/MWh for small consumers and 10 EUR/MWh for industries.

Energy taxes in Sweden by 2020 are 35 öre/kWh for private consumers and small industries, 0.5 öre/kWh for heavy industries. A VAT of 25% is applied to the sum of electricity price, grid fee, and an energy tax. In this study, the profit (cost) function for consumers includes grid fees, energy taxes (converted to EUR/MWh), and VAT.

***Biomass price***

The price of biomass (pellets) affects the costs associated with the heating sector that is not supplied by district heating (DH). Swedish annual average pellet prices from 2010 to 2018 range between 34.39 EUR/MWh in 2017 and 38.03 EUR/MWh in 2011 [34] (including delivery for household supply). The biomass price was set to 37 EUR/MWH for this study.

In summary, the consumers profit (cost) or welfare function includes technology costs *C_ann_* electricity costs and grid fees, fuel costs (pellets) and respective taxes. In cases where energy efficiency measures or biomass boilers are a favorite choice, such investment costs are included. Other additional costs to the consumers arise depending on the implemented policy.

**References**

[1] Micropolis. Arctic Energy - Energiaomavarainen pohjoinen - Greenpolis. Micropolis 2019. https://www.greenpolis.fi/en/projektit/arctic-energy/ (accessed May 16, 2019).

[2] Fischer R, Elfgren E, Toffolo A. Towards optimal sustainable energy systems in Nordic municipalities. Energies 2020;13. https://doi.org/10.3390/en13020290.

[3] Liljeblad A. Framtidens elanvändning. En delrapport. Stockholm: 2016.

[4] LKAB, SSAB, Vattenfall. Fossil-free Steel - Hybrit. HYBRIT, Foss Steel 2016. http://www.hybritdevelopment.com/ (accessed June 7, 2019).

[5] Swedish Energy Agency. Energiläget i siffror 2019. Energimyndigheten 2019. http://www.energimyndigheten.se/nyhetsarkiv/2019/Nu-finns-siffror-pa-energilaget-i-Sverige/ (accessed May 10, 2019).

[6] Wikman Å. Klimat-och energiplan Piteå. Piteå: 2010.

[7] Statisticon AB. Befolkningsprognos 2018-2030 Piteå kommun. Uppsala: 2018.

[8] Konjunkturinstitutet. Prognosjämförelse. Konjunkturinstitutet 2019. https://www.konj.se/publikationer/konjunkturlaget/prognosjamforelse.html (accessed June 27, 2019).

[9] SCB. Statistikdatabasen. SCB 2019. http://www.statistikdatabasen.scb.se/ (accessed May 15, 2019).

[10] Energikontor Norr. Energiluppen. Energikontor Norr 2019. http://www.energiluppen.se/ (accessed May 15, 2019).

[11] Piteå Kommun. Piteå climate and energy plan 2010. Supporting documents 2016.

[12] Piteå industries. Energy data of local industries in Piteå 2016.

[13] PiteEnergi AB. Årsredovisning och koncernredovisning för AB PiteEnergi, Räkenskapsåret 2015. PIteå: 2016.

[14] Miljö- och energidepartementet. Miljöbalk (1998:808). Stockholm: Sveriges riksdag; 1999.

[15] Swedish Energy Agency. Ny samlingspublikation: energistatistik för småhus, flerbostadshus och lokaler 2013. Swedish Energy Agency 2014. http://www.energimyndigheten.se/nyhetsarkiv/2014/ny-samlingspublikation-energistatistik-for-smahus-flerbostadshus-och-lokaler-2013/ (accessed May 15, 2019).

[16] Swedish Energy Agency. Bostäder och lokaler. Swedish Energy Agency 2016. http://www.energimyndigheten.se/statistik/bostader-och-lokaler/ (accessed May 15, 2019).

[17] Steinbach J, Isi F, Staniaszek D. Discount rates in energy system analysis Discussion Paper. 2015.

[18] García-Gusano D, Espegren K, Lind A, Kirkengen M. The role of the discount rates in energy systems optimisation models. Renew Sustain Energy Rev 2016;59:56–72. https://doi.org/10.1016/j.rser.2015.12.359.

[19] Hartman RS, Doane MJ. Household Discount Rates Revisited. Energy J 1986;7.

[20] Schleich J, Gassmann X, Faure C, Meissner T. Making the implicit explicit: A look inside the implicit discount rate. Energy Policy 2016;97:321–31. https://doi.org/10.1016/j.enpol.2016.07.044.

[21] Aalborg University. EnergyPLAN | Advanced energy systems analysis computer model. Dep Dev Planning, Aalborg Univ 2019. https://www.energyplan.eu/ (accessed May 13, 2019).

[22] Danish Energy Agency, Energinet. Technology Data. Danish Energy Agency 2019. https://ens.dk/en/our-services/projections-and-models/technology-data (accessed May 14, 2019).

[23] Tsiropoulos I, Tarvydas D, Zucker A. Cost development of low carbon energy technologies. 2018. https://doi.org/10.2760/490059.

[24] Moles C, Sigfusson B, Spisto A, Vallei M, Weidner E, Giuntoli J, et al. Energy Technology Reference Indicator (ETRI) projections for 2010-2050. 2014.

[25] Swedish government. Sweden’s Fourth National Energy Efficiency Action Plan. Stockholm: 2014.

[26] Hirvonen J, Jokisalo J, Heljo J, Kosonen R. Towards the EU emissions targets of 2050: optimal energy renovation measures of Finnish apartment buildings. Int J Sustain Energy 2019;38:649–72. https://doi.org/10.1080/14786451.2018.1559164.

[27] Hirvonen J, Jokisalo J, Heljo J, Kosonen R. Towards the EU emission targets of 2050: Cost-effective emission reduction in Finnish detached houses. Energies 2019;12. https://doi.org/10.3390/en12224395.

[28] Discher H, Hinz E, Enseling A. dena-Sanierungsstudie. Teil 1: Wirtschaftlichkeit energetischerModernisierung im Mietwohnungsbestand. Berlin: 2010.

[29] Stolte C, Marcinek H, Discher H, Hinz E, Enseling A. dena-Sanierungsstudie. Teil 2: Wirtschaftlichkeit energetischer Modernisierung in selbstgenutzten Wohngebäuden. Berlin: 2011.

[30] Shadram F, Bhattacharjee S, Lidelöw S, Mukkavaara J, Olofsson T. Exploring the trade-off in life cycle energy of building retrofit through optimization. Appl Energy 2020;269. https://doi.org/10.1016/j.apenergy.2020.115083.

[31] NORDPOOL. Historical Market Data. Nordpool 2019. https://www.nordpoolgroup.com/historical-market-data/ (accessed May 13, 2019).

[32] Energiföretagen. Spotprisets utveckling - Energiföretagen Sverige 2022. https://www.energiforetagen.se/energifakta/elsystemet/elhandel/spotprisets-utveckling/ (accessed June 3, 2022).

[33] Energimarknadsinspektionen. Nätavgifter - elnät - Energimarknadsinspektionen. Energimarknadsinspektionen n.d. https://www.ei.se/sv/statistik/statistik-inom-omradet-el/Statistik-om-elnat/elnatsavgifter/ (accessed August 26, 2021).

[34] Pelletsförbundet. Statistik | Pelletsförbundet. Pelletsförbundet 2019. http://pelletsforbundet.se/statistik/ (accessed May 14, 2019).
