# Supplementary material for: Game theory-based analysis of policy instrument consequences on energy system actors in a Nordic municipality
Source: Heliyon. 2024 Feb 4;10(4):e25822. doi: 10.1016/j.heliyon.2024.e25822 (PMC10881334; doi:10.1016/j.heliyon.2024.e25822)
Supplement: Multimedia component 2 [file mmc2.docx]

**Game theory-based analysis of policy consequences on energy system actors in a Nordic municipality**

Robert Fischer^*,a^, Andrea Toffolo^a^

^a^ Energy Engineering, Division of Energy Science, Luleå University of Technology, SE-97187 Luleå.

^*^ Corresponding author: [robert.fischer@associated.ltu.se](mailto:robert.fischer@associated.ltu.se)

**Supplementary material, Part B**

**Additional investigations, results and analysis**

The supplementary material Part B provides additional information about the investigated policy instruments and includes parameters and cases that have been tested, though not explicitly presented in the article.

Content

[Key parameters 3](#_Toc120862723)

[Multi-objective optimization and game theory approach 3](#_Toc120862724)

[Base cases 4](#_Toc120862725)

[Base case, analysis of game theory solutions 5](#_Toc120862726)

[CO2-tax on consumers, protecting the industry 6](#_Toc120862727)

[CO2-tax on consumers, protecting the industry, analysis of game theory solutions 7](#_Toc120862728)

[Electricity certificate scheme (ELcert-scheme) 9](#_Toc120862729)

[ELcert-scheme, analysis of game theory solutions 10](#_Toc120862730)

[Contract for Difference (CfD) 11](#_Toc120862731)

[CfD, analysis of game theory solutions 12](#_Toc120862732)

[Investment subsidies to Consumers 13](#_Toc120862733)

[References 15](#_Toc120862734)

# Key parameters

Electricity suppliers (utilities) and consumers are independent interacting actors in the electricity and heating energy sectors. A consumer group with heating demand from buildings not connected to district heating significantly contributes to electricity use in Nordic municipalities. A local municipal utility and this specific consumer group are the investigated actors in this study. Policy instruments are required to enable market actors to contribute to GHG emission reduction targets and distribute costs and benefits fairly between them. The consequences of selected policy instruments are analyzed and discussed in this study. The methodical approach includes modeling the energy system of a representative municipal energy system (Piteå, Norrbotten, Sweden) in the advanced simulation tool EnergyPLAN. The local vertically integrated electricity supplier (the Utility or “U”) and the specific heating consumer group not connected to district heating (the Consumers or “C”) are described in detail in the main article.

The energy system model of Piteå, including technical and economic parameters for renewable energy, heating technologies, and energy efficiency (*EE*) measures, are described in the supplementary material Part A. This study is interested in achieving a 50% emission reduction of the total annual CO2 emissions of 56.675 ktCO2/year, which result from grid electricity import, by applying and investigating several policy instruments on the two actors.

Key parameters and assumptions applied in the studied cases and sensitivity analysis are listed in Table 1:

Table 1: Key parameters used in the Base case and the studied policy instrument cases

| **Parameter description** | **Base case value** | **Investigated values** |
| --- | --- | --- |
| Annual average electricity spot price: ESP [EUR/MWh] | 25 EUR/MWh  (Considering historical values and futures on NASDAQ as of September 2020) | 40 EUR/MWh (high electricity price scenario)  International markets increasingly impact Nordic electricity prices. |
| Biomass (pellets) prices delivered to the doorstep: | 7.28 EUR/MWh + 3 EUR/MWh transport costs. | Variations in biomass prices are assumed to be insufficient to change consumers´ choices. |
| Discount rate Utility (*dru*) | 9% (representing WACC+1.5%) | 5% (widely used in ESOM, e.g., in EU-TIMES)  7.5% (representing WACC) |
| Discount rate Consumers (*drc*) | 3% | Not applicable. |
| CO2-tax [EUR/tCO2] | 0 EUR/tCO2 | 30 EUR/tCO2 (recommended by e.g., [1])  100 EUR/tCO2 (current Swedish level [2]) 200 and up to 400 EUR/tCO2 (levels as recommended by, e.g., Nordhaus [3]) |
| Electricity certificate scheme; price for electricity certificate [EUR/MWh] | 0 EUR/MWh | 1.5, 5, 10, and 20 EUR/MWh |
| Grid emission factor [tCO2/MWh] | 0.156 (assumed as Nordic average) | Sweden 0.016; Finland 0.156; Denmark = 0.333. EU28=0.393 |
| Investment grants for *BioB* and *EE measures* | 0% | 25%; 50%; 75% |

# Multi-objective optimization and game theory approach

The Utility profit and the Consumers’ cost function are the objective functions. EnergyPLAN is interfaced with a multi-objective optimization implemented as an evolutionary algorithm in Matlab; Pareto optimal solutions are then subjected to game theory-based analysis to understand the policy consequences and the results of cooperative and non-cooperative behavior of the selected actors.

Technical parameters for the multi-objective optimization with an evolutionary algorithm include the number of individuals, the number of generations, and the mutation probability (0.05). In most studied cases, the parameters for individuals and generations are set to 200. Some cases are tested only with 100 individuals and generations, while others require more generations to achieve a satisfying consolidation of final Pareto front (PF).

In the following, matrices of diagrams are used to organize the results from the multi-objective optimization showing:

- the Pareto front (“Costs”) between the two objective functions, Utility profits ([MEUR], x-axis, axis titles are omitted) and Consumers costs (“HTindi” [MEUR], primary y-axis), shown in both rows of diagrams;
- installed RE capacities ([MW], secondary y-axis) for Solar PV (“PV”), onshore wind power (“WindON”) and offshore wind power (“WindOFF”), shown in the first row of diagrams;
- the shares of the annual heating demand ([GWh], secondary y-axis) satisfied by biomass boilers (“BioB”), heat pumps (“HP”) and direct electric heating in the form of electric boilers (“ElB”), shown in the second row of diagrams;
- total CO2 emissions (“CO2-tot” [ktCO2], primary y-axis), shown in the first row of diagrams.

The game theory-based approach, including the analysis of the Nash equilibrium (NE) for the non-cooperative game (which characterizes an outcome in which no player can increase their gain by unilaterally modifying its strategy given the strategies chosen by the other players), the disagreement outcome (DO) and the Nash bargaining solution (NBS) for the cooperative game, is described in the main article.

# Base cases

The Base cases investigates ESPs of 25 EUR/MWh and 40 EUR/MWh. Investigated Utility discount rates *dru* are a social discount rate of 5% as used widely in energy systems analysis, 7.5% (WACC) and 9%, representing the WACC+1.5%. The Consumers discount rate *drc* is 3%, a commonly applied discount rate for households. Research, however, shows that households with low incomes can apply much higher discount rates (30%) on energy investments as disposable income serves other priorities.

The Base cases with ESP 25 and 40 EUR/MWh, *dru* 9%, 7.5%, and 5%, clearly show the LCOE relationship to the ESP (Figures 1 to 3), resulting in either no investment or maximum investment in onshore windpower as ESP is lower or higher than the respective LCOE (LCOEs are affected by *dru*, as presented in Table 2). Solar PV becomes a convenient investment and reaches maximum capacity with the higher ESP and a *dru* of 5%. The Consumers´ preferences are not visibly affected by any of these parameter ranges as investing in *HP* is the most convenient (cheapest) heating option in all cases. The CO2 emission reduction target of 50% is reached only in the cases in which the Utility finds it convenient to invest in new RE.

In the investigated policy instruments the discount rate for Consumers (*drc*) was kept at 3%. Discount rates for the utility (*dru*) are varied in some cases, as in the Base case. A higher ESP of 40 EUR/MWh is applied in some cases.

Table 2: LCOE with different Utility discount rates dru

|  | LCOE [EUR/MWh] | | |
| --- | --- | --- | --- |
| *dru* | Solar PV | Onshore wind | Offshore wind |
| 9% | 50.3 | 40.0 | 68.1 |
| 7.5% | 44.5 | 36.3 | 61.1 |
| 5% | 35.6 | 30.5 | 50.2 |


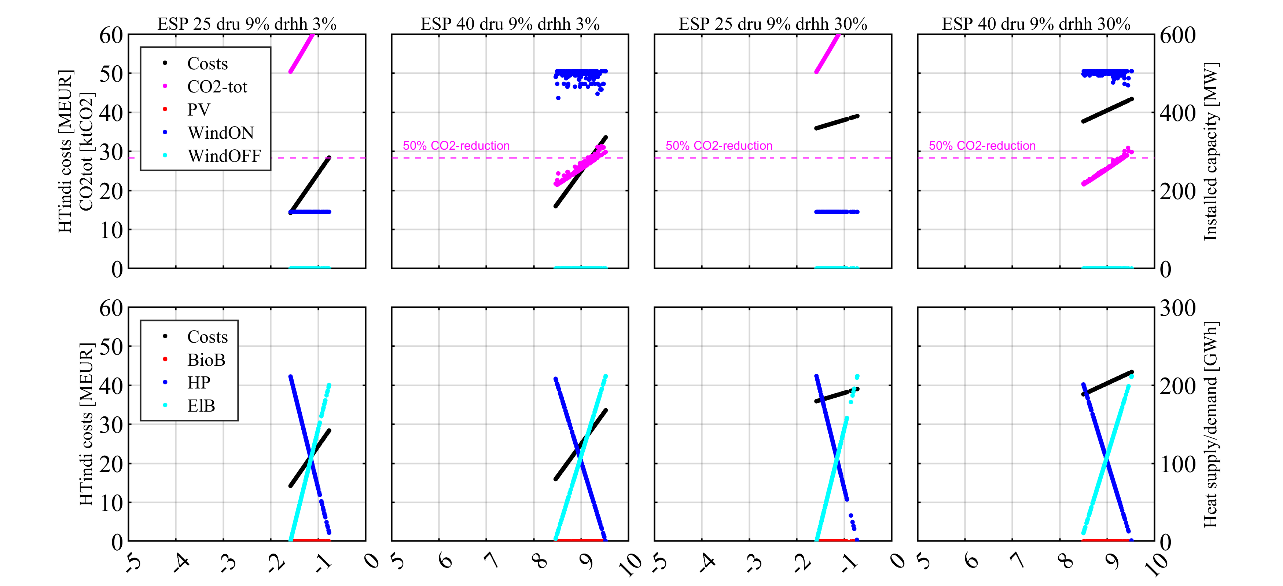


Figure 1: Base case, ESP=25 or 40 EUR/MWh; dru=9%; drc=3% or 30%


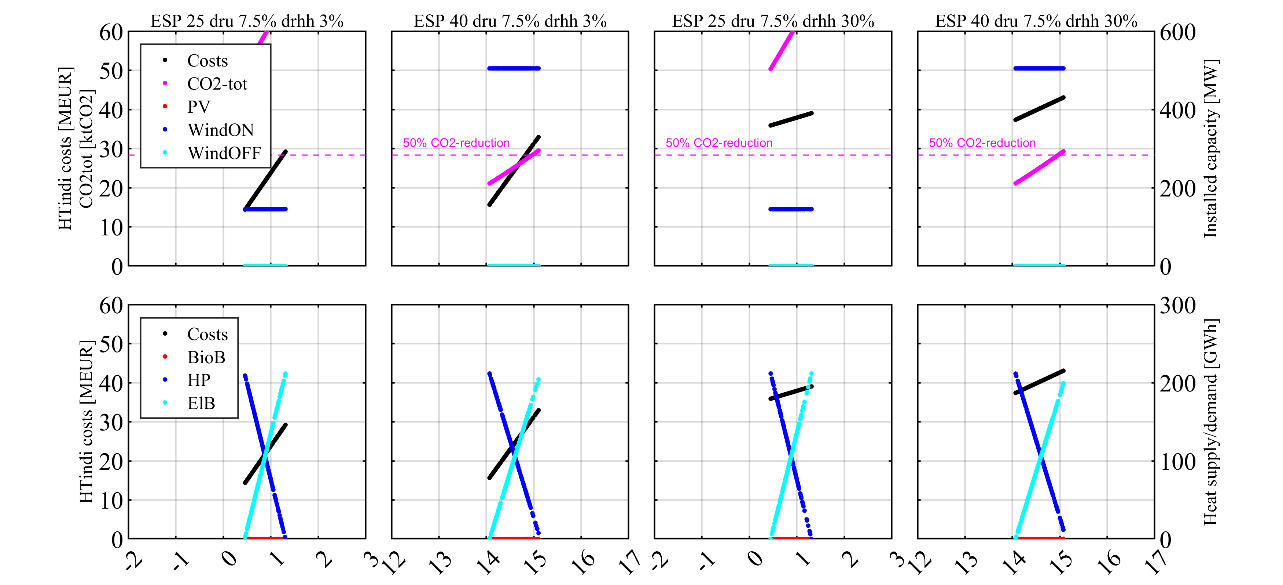


Figure 2: Base case, dru=7.5%; cet. par.


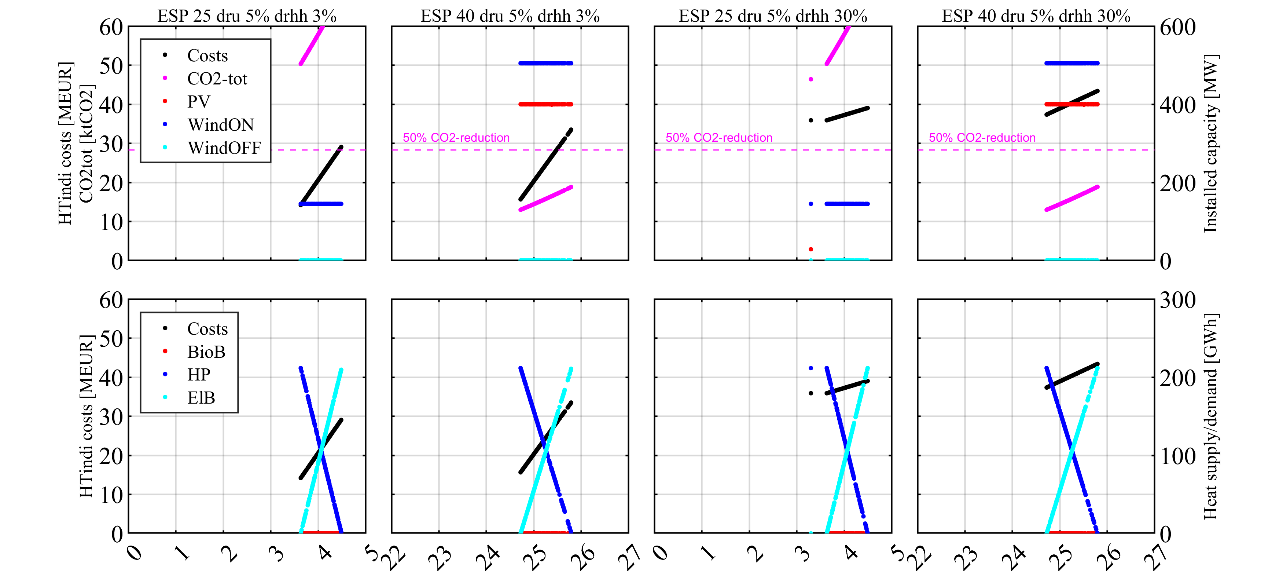


Figure 3: Base case, dru=5%; cet. par.

### Base case, analysis of game theory solutions

In all the Base case variations, the same situation is observed. The Consumers’ strategy determines the electricity demand, affecting Utility profits. Higher demand from the Consumers result in higher Utility sales (and profit), but also in higher Consumers costs. On the other hand, the Utility strategy does not affect Consumers’ costs. For a 25 EUR/MWh ESP, the Utility will not invest, as it would result in lower profits, and therefore it has only one best strategy - not to invest - regardless of what the Consumers choose. While the Utility has a single best strategy whatever the Consumers do, the Consumers have just to lower their costs, which is possible by investing in heat pumps (*HP*).

Accordingly, the NE is the leftmost point of the PF, and, being a point on the PF, it also coincides with the NBS. This is because in the cooperative game the DO is the NE itself, so the closest point on the PF according to the hyperbole of the excess *payoffs* (the NBS), coincides with the same point. The players´ *payoffs* for the Base case with a 25 EUR/MWh ESP are presented in a simplified version of the generalized form for the strategic game in Table 3, where higher and lower *payoffs* of the two players (Utility and Consumers, U and C) are represented with plus and minus signs. The cell which locates the NE is marked with a thick border.

Table 3: Payoffs for the Base case – no policy instrument with ESP=25 EUR/MWh

|  | | Consumers | |
| --- | --- | --- | --- |
|  |  | *HP* | *EB* |
| Utility | invest | U- C+ | U- C- |
|  | no invest. | U+ C+ | U+ C- |

An analysis of the payoffs in the neighborhood of a generic point on the PF is shown in Table 4 and Figure 4, marking with “0” the reference payoffs for the considered point and higher and lower *payoffs* with plus and minus signs. The analysis shows that a generic point on the PF (Figure 4) is not a NE because Consumers can find a better strategy by using more HP when the Utility does not invest. The only NE is found when Consumers cannot use more HP (already 100% HP). This is also the NBS (because it is on the PF).

Table 4: Analysis of a generic point on the PF for the Base case with ESP=25 EUR/MWh

|  | | Consumers | | |
| --- | --- | --- | --- | --- |
|  |  | more HP | as in PF | more EB |
| Utility | invest more |  | U- C0 |  |
|  | no invest. (as in PF) | U- C+ | U0 C0 | U+ C- |

Consumers costs


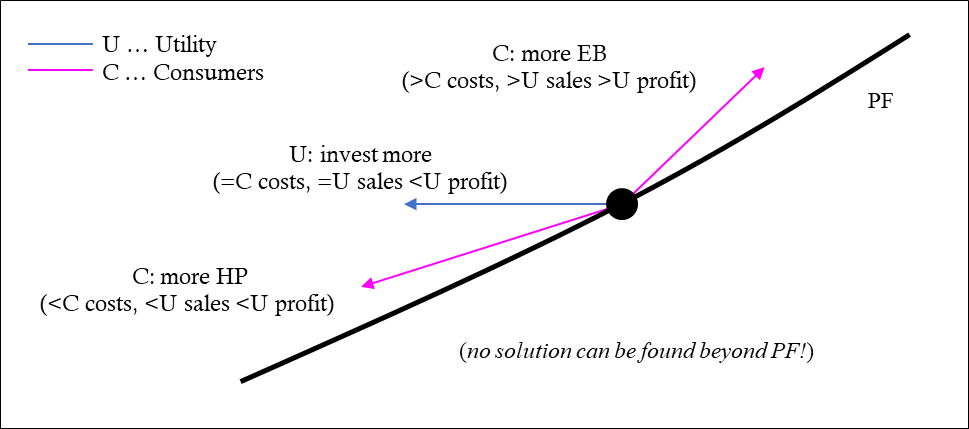


Utility profit

Figure 4: Analysis of a generic point on the PF for the Base case with ESP=25 EUR/MWh

# CO2-tax on consumers, protecting the industry

This section analyzes the results of the CO2-tax policy instrument applied to non-industrial consumers. The investigated CO2-tax rates (“co2taxcnoind” in the diagrams) are 30, 100, 200 (not shown in the diagrams), 400 and 800 EUR/tCO2. The total annual CO2 cost to be paid by the electricity consumers within the modeled scope is determined by the imported grid electricity (GWh/year) and its related annual CO2 emissions (tCO2/year), which is multiplied by the CO2-tax rate. The total CO2 cost is then converted to a CO2 cost per kWh for the electricity consumers. The accrued CO2-tax is a revenue to the government.

Below a CO2-tax rate of about 400 EUR/tCO2, the Consumers’ best choice is to invest in *HP*. For that rate and above, the best option is to invest in *BioB* instead, further reducing emissions due to reduced electricity use and import from the grid (Figure 5 and Figure 6). A CO2-tax applied under the higher electricity price scenario (ESP=40 EUR/MWh), cet. par., results in the same Consumers’ choices, but the Utility finds it convenient to invest in onshore wind power (as in the Base case, of course, for the same ESP).


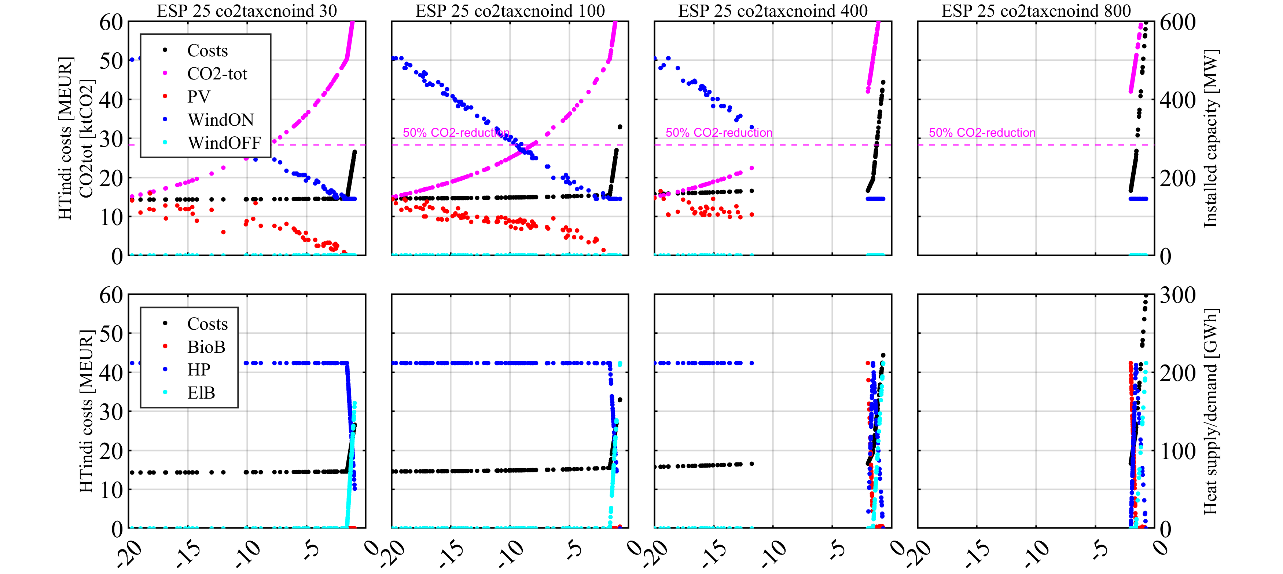


Figure 5: CO2-tax on consumers, protecting industry. ESP=25 EUR/MWh. CO2-tax rates=30, 100, 400, 800 EUR/tCO2


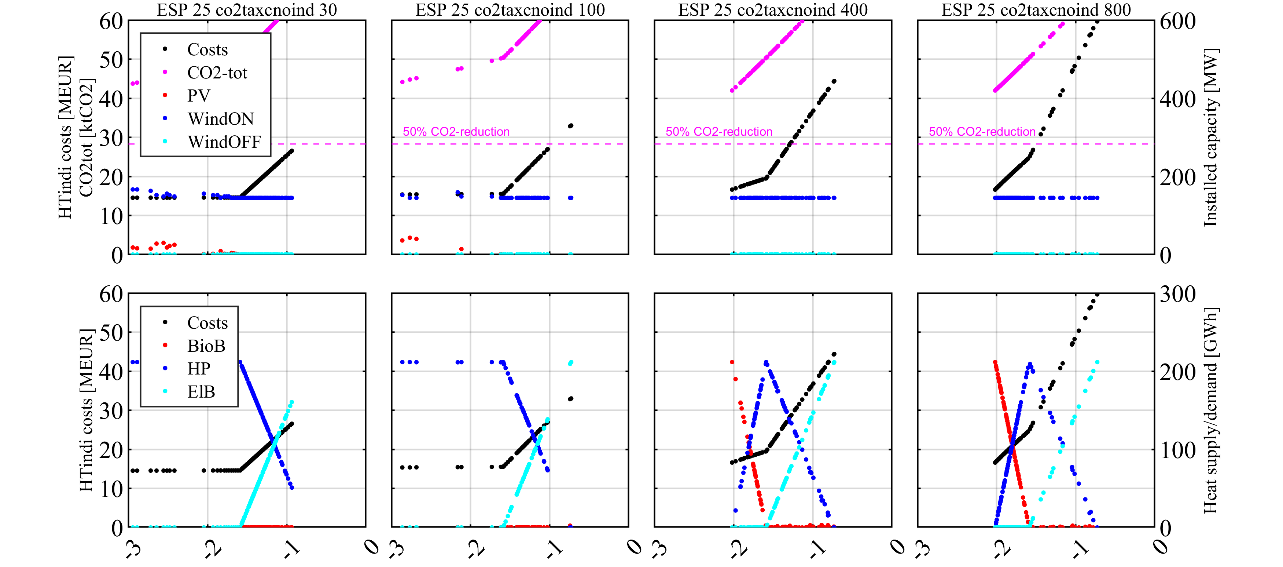


Figure 6: CO2-tax on consumers, protecting industry. ESP=25 EUR/MWh. CO2-tax rates=30, 100, 400, 800 EUR/tCO2 (x-axis zoomed in)

### CO2-tax on consumers, protecting the industry, analysis of game theory solutions

The situation with the CO2-tax on Consumers, protecting the industry, is somewhat similar to the Base case. The Utility will not invest just to decrease the costs for the Consumers (it would only increase losses since the ESP is lower to the LCOEs of RE technologies), so the wide leftmost, almost horizontal part of the PF in Figure 5 should be disregarded in the game theory analysis, although Pareto optimal. The Consumers will do their best to lower their costs while the utility does not invest, so they will use 100% *HP* unless the CO2-tax is so high that *BioB* becomes even more convenient.

There is only one NE (which is also DO and NBS), i.e. the cheapest option for the Consumers (*HP* or *BioB*, depending on the CO2-tax rate) while the utility does not invest. An analysis of a generic point on the right part of the PF in column 2 of Figure 6 is shown in Table 5 and Figure 7, marking with “0” the reference payoffs for the considered point and higher and lower *payoffs* with plus and minus signs

Table 5: Analysis of a generic point on the PF for the CO2-tax case (rightmost part), protecting the industry, ESP=25 EUR/MWh, CO2-tax 100 EUR/tCO2

|  | | Consumers | | |
| --- | --- | --- | --- | --- |
|  |  | more HP | as in PF | more EB |
| Utility | invest more |  | U- C+ (1) |  |
|  | no invest (as in PF) | U- C+ (2) | U0 C0 | U+ C- (3) |

Starting from the considered point, the following strategies are available to the two players:

1. Utility invests more: Electricity demand stays constant, supply increases, import decreases. Consumers’ costs are lower (less CO2-tax thanks to lower imports). Utility profit is lower (higher losses because LCOE is lower than ESP).
2. Consumers more *HP*: Lower demand, supply constant, less import. Consumers’ costs are lower (both lower electricity costs and less CO2-tax). Utility profit is lower (lower sales).
3. Consumers more *EB*: Higher demand, supply constant, higher import. Consumers’ costs are higher (more electricity costs and more CO2-tax). Utility profit is higher (higher sales).

Accordingly, a generic point on the right part of the PF in column 2 of Figure 6 is not a NE because Consumers can find a better strategy while the Utility does not invest (2).

Consumers costs


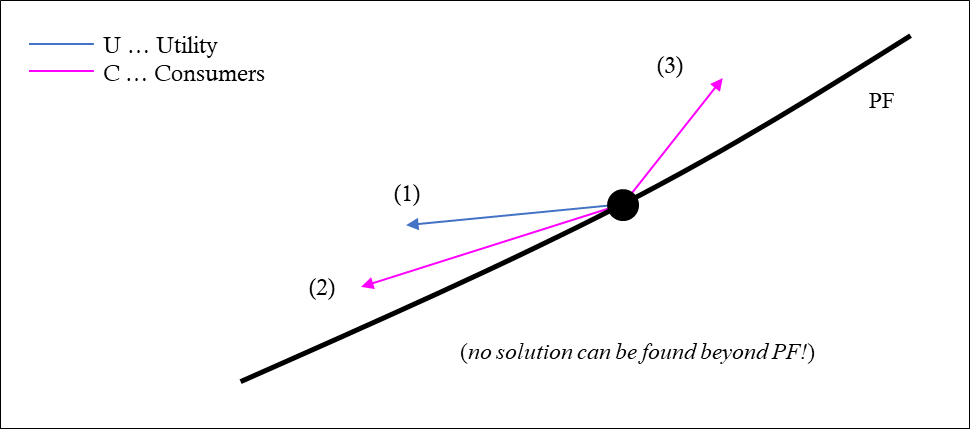


Utility profit

Figure 7: Analysis of a generic point on the PF for the CO2-tax case (rightmost part), protecting the industry, ESP=25 EUR/MWh, CO2-tax 100 EUR/tCO2

A similar analysis for a generic point on the leftmost part of the PF in Figure 5 (which actually can be disregarded due to high losses for the Utility, but the solutions are still Pareto optimal!) is shown in Table 6 and Figure 8):

Table 6: Analysis of a generic point on the PF for the CO2-tax case (leftmost part), protecting the industry, ESP=25 EUR/MWh, CO2-tax 100 EUR/tCO2

|  | | Consumers | |
| --- | --- | --- | --- |
|  |  | 100% HP as in PF | some EB |
| Utility | invest more | U- C+ (2) |  |
|  | as in PF | U0 C0 | U+ C- (1) |
|  | invest less | U+ C- (3) |  |

Starting from the considered point, the following strategies are available to the two players:

1. C invests in *EB*: Electricity demand is higher, supply constant, higher import. Consumers’ costs are higher (more heating costs and CO2-tax for higher imports). Utility profit is higher (higher sales).
2. U invests more: Same demand, supply increases, lower import. Consumers’ costs are lower (same heating costs and less CO2-tax). Utility profit is lower (higher losses because LCOE is lower than ESP).
3. U invests less: Same demand, supply decreases, higher import. Consumers’ costs are higher (same heating costs but more CO2-tax). Utility profit is higher (lower losses).

Accordingly, a generic point on the leftmost part of the PF is not a NE because the Utility can find a better strategy (until investments are zero) when C chooses 100% HP (3).

Consumers costs


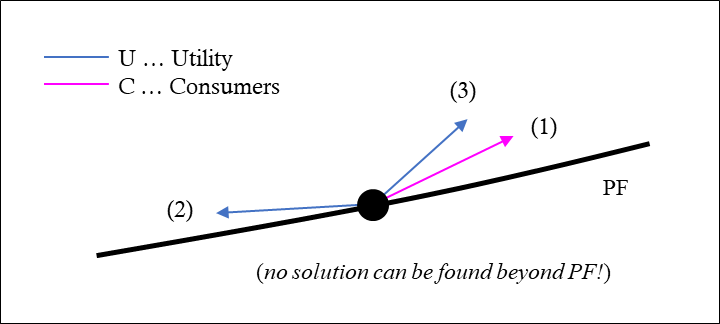


Utility profit

Figure 8: Analysis of a generic point on the PF for the CO2-tax case (leftmost part), protecting the industry, ESP=25 EUR/MWh, CO2-tax 100 EUR/tCO2

# Electricity certificate scheme (ELcert-scheme)

The electricity certificate scheme (ELcert-scheme) was introduced in Sweden in 2003, Norway joined in 2010, and will end in 2035. The scheme is a market-based support system and provides financing in the form of certificates for each produced MWh of eligible renewable technologies. A legally established quota system creates and guarantees a market where these certificates are traded, and the ELcert-price is established (Figure 9).


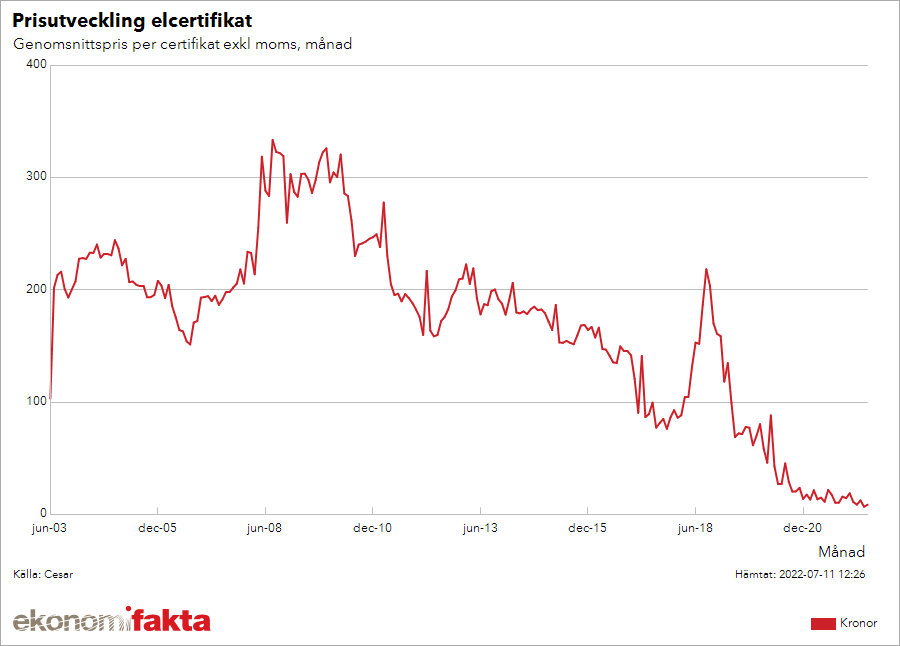


Figure 9: ELcert-scheme: historical price development of the electricity certificate [SEK/MWh] (Source: Ekonomifakta)

Electricity traders and consumers are obliged to buy such certificates according to the current quota and pay according to a statistically derived cost; a simplified formula for the ELcert-cost to the consumer is [4]:

ELcert-cost = ELcert-price * quota.

For 2020 the ELcert-cost was 1.8 öre/kWh, or about 1.8 EUR/MWh for the consumer; future ELcert-prices are expected to be even lower as the electricity market is oversupplied due to a higher than envisioned increase in windpower capacity. Windpower growth is expected to continue strongly, both onshore and offshore. Solar PV is also growing fast but is still far away from a significant market share.

The ELcert policy instrument case implements ELcert-prices of 1.5, 5, 10, and 20 EUR/MWh and a quota of 26.6 in 2020 (Figure 12). With an ESP of 25 EUR/MWh, an ELcert-price of about 20 EUR/MWh would be required to compensate for the utility losses when investing in onshore wind with an LCOE of 40 EUR/MWh (column 4 in Figure 10). With an ELcert-price of 30 EUR/MWh, even Solar PV would become convenient for the Utility to invest in. The additional cost for the consumer due to the ELcert-cost is insignificant and does not affect the Consumers’ technology choices. The presented ELcert cases in Figure 12 provide an ELcert-revenue to already installed *windON* and to new *windON*, resulting in increasing profits with increasing ELcert-prices, even without new capacities. The available ELcert-volume is also not limited by the geographical scope, as compared to the implementation presented in the main article, resulting in the Utility investing up to the maximum possible *windON* capacity for ELcert-price of 20 EUR/MWh.


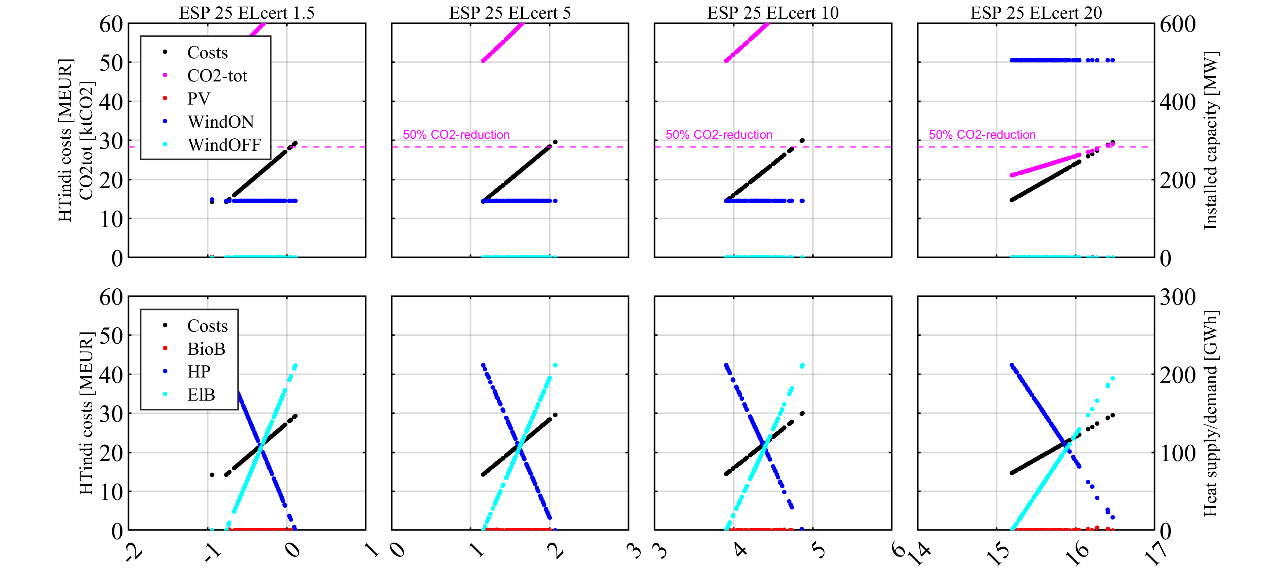


Figure 10: Electricity certificate scheme. ESP=25 EUR/MWh.

### ELcert-scheme, analysis of game theory solutions

An ELcert-cost term is added to the Consumers profit (cost) function, and an ELcert revenue term is added to the Utility profit function. As ELcert-prices increase, Utility profit grows and Consumers´ costs too. The Utility is compensated with the ELcert for already installed onshore wind, hence a profit increase. However, no new investments can be observed for ELcert levels below 20 EUR/MWh (columns 1-3 in Figure 10).

The game appears to be identical to the Base case game. Table 7 and Figure 11 show the analysis of a generic point on the PF for ELcert-price below 20 EUR/MWh (columns 1-3 in Figure 12), marking with “0” the reference payoffs for the considered point and higher and lower *payoffs* with plus and minus signs.

Table 7: Analysis of a generic point on the PF for the ELcert scheme (ELcert-price below 20 EUR/MWh)

|  | | Consumers | | |
| --- | --- | --- | --- | --- |
|  |  | more HP | as in PF | more EB |
| Utility | invest more |  | U- C- (1) |  |
|  | no invest (as in PF) | U- C+ (2) | U0 C0 | U+ C- (3) |

Starting from the considered point, the following strategies are available to the two players:

1. U invest more: Electricity demand is the same, supply increases, import decreases. The Utility gets more revenues from ELcert, but not sufficient to cover the losses, so its profit is lower. Consumers’ costs slightly increase as they pay more for ELcert in bill.
2. C more HP: less demand, same supply, import decreases. Utility profit decreases (lower electricity sales, same ELcert). Consumers’ costs decrease (lower heating costs, lower ELcert in the bill).
3. C more EB: higher demand, same supply, import increases, Utility profit increases (higher electricity sales, same ELcert). C costs increase (higher heating costs, higher ELcert in the bill).

Consumers costs


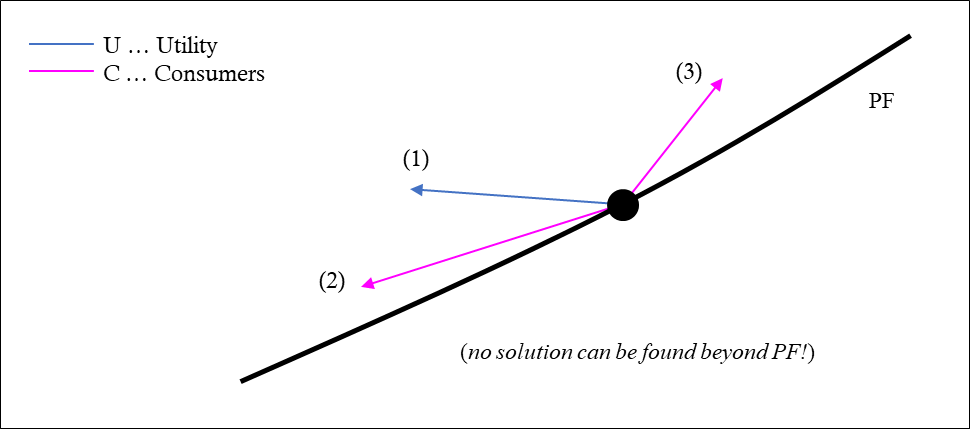


Utility profit

Figure 11: Analysis of a generic point on the PF for the ELcert scheme (ELcert-price below 20 EUR/MWh)

# Contract for Difference (CfD)

CfD is an auction-based policy instrument that finances new renewable electricity generation by closing the gap between the ESP and a Strike Price (in this study, the Strike Price is equal to the LCOE). A CO2-tax (or fee) finances the CfD on consumers (protecting the industry), in a similar way to the CO2-tax policy instrument implemented in this paper. The difference is that the accrued CO2-tax is not passed to the government, but it is used as the available CfD budget.

CO2-tax levels of 30, 100, 200, and 400 EUR/tCO2 are applied and create a limited CfD budget, which depends on electricity import-related CO2 emissions. The results of the multi-objective optimization are presented in Figure 12 and Figure 13.


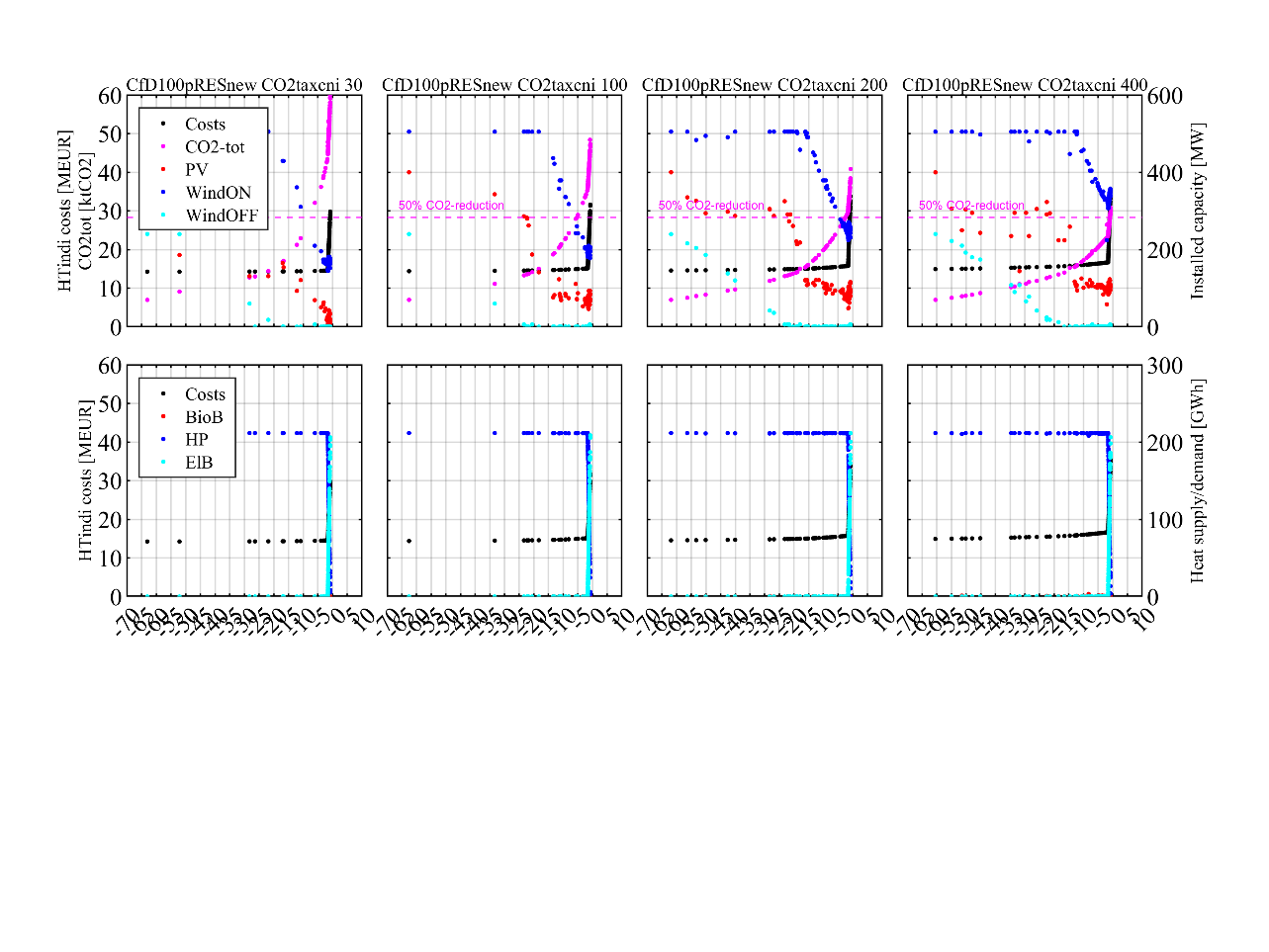


Figure 12: CfD with CO2-tax rates = 30, 100, 200 and 400 EUR/tCO2.


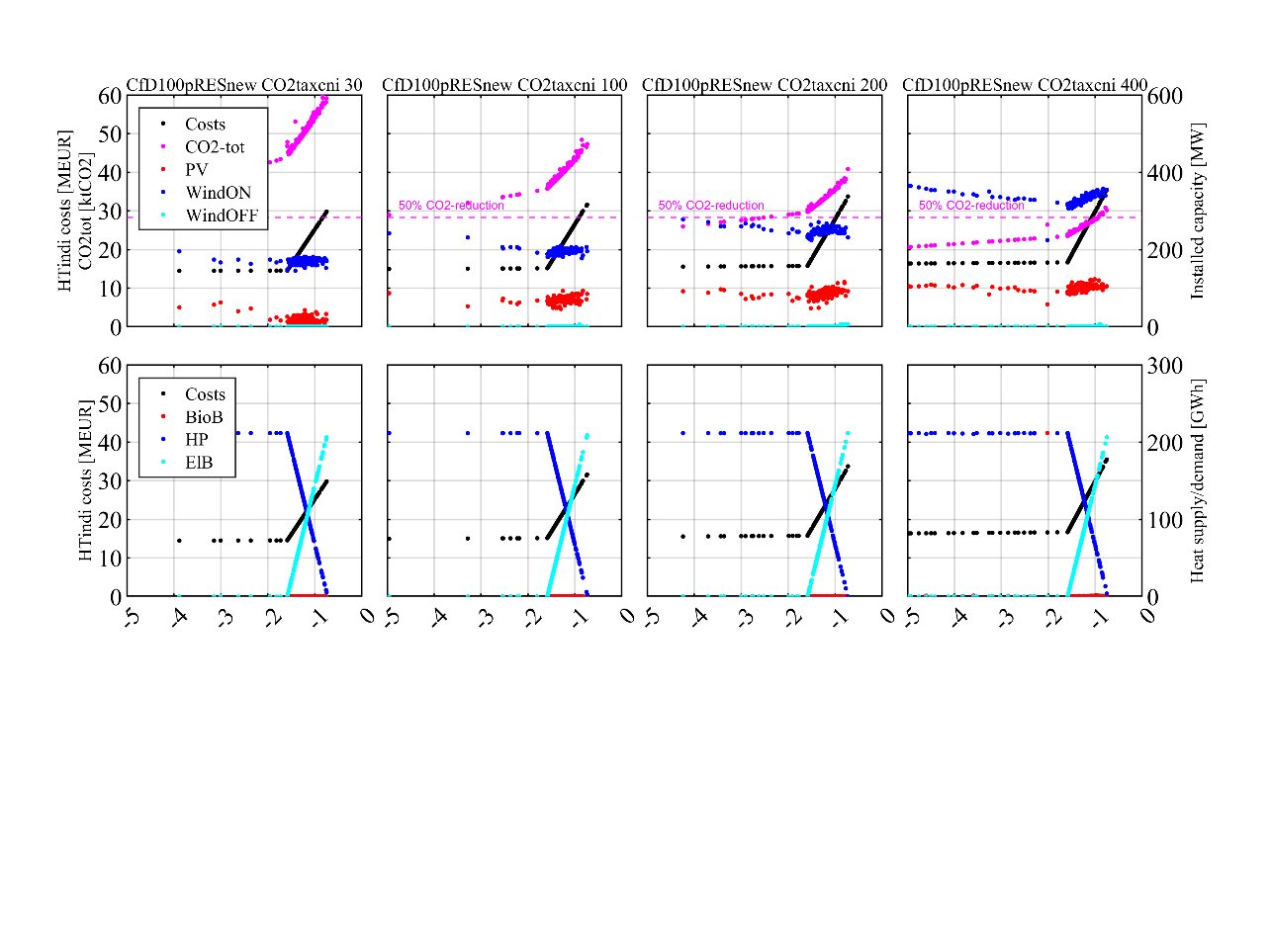


Figure 13: CfD with different CO2-tax rates (x-axis zoomed in)

### CfD, analysis of game theory solutions

The strategy chosen by the Utility determines the supply (availability) of electricity from local generation and subsequently the total CO2-tax to be paid; the strategy chosen by Consumers determines the electricity demand. Table 8 analyses a generic point on the PF for CfD with a CO2-tax of 200 EUR/tCO2 (column 3 in Figure 12 and Figure 13), marking with “0” the reference payoffs for the considered point and higher and lower *payoffs* with plus and minus signs.

Table 8: Analysis of a generic point on the PF for CfD and CO2-tax = 200 EUR/tCO2.

|  | | Consumers | | |
| --- | --- | --- | --- | --- |
|  |  | more HP | as in PF | more EB |
| Utility | invest more |  | U- C+ (3) |  |
|  | as in PF | U- C+ (1) | U0 C0 | U0+ C- (2) |
|  | invest less |  | U0+ C- (4) |  |

Starting from the considered point, the following strategies are available to the two players:

1. Consumers use more HP and less EB: lower electricity demand due to more HP and same local supply (Utility investment remain as in PF) result in less import and less CO2-tax. Consumer costs are lower (lower heating costs, lower CO2-tax), Utility profit is worse (lower money transfer from CfD).
2. Consumers use more HP and less EB: higher electricity demand due to more EB and same local supply (Utility investment remain as in PF) result in more import and more CO2-tax. Consumer costs are higher (higher heating costs, higher CO2-tax), Utility profit could increase if the higher money transfer from CfD mechanism is matched by Utility investments.
3. Utility invests more: Same electricity demand as in PF and more local supply result in less import and less CO2-tax. Consumer costs are lower (same heating costs but lower CO2-tax), Utility profit is worse (lower money transfer from CfD).
4. Utility invests less: Same electricity demand as in PF and less local supply result in more import and more CO2-tax. Consumer costs are higher (same heating costs but higher CO2-tax), Utility profit could increase due to lower production costs and the higher money transfer from CfD mechanism (if matched by Utility investments).


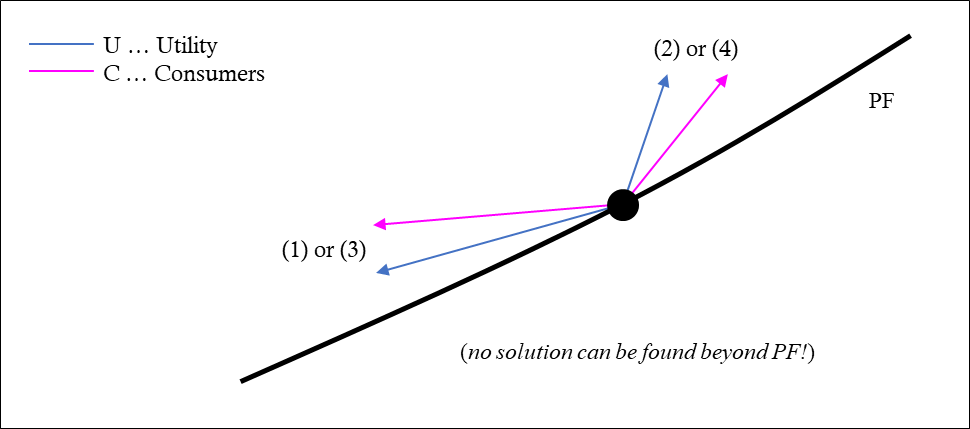


Utility profit

Consumers costs

Figure 14: Analysis of a generic point on the PF for CfD and CO2-tax = 200 EUR/tCO2

None of the points on the PF is a NE because both Utility and Consumers can find better strategies than the PF strategy while the other player keeps its own. However, a NE can be found inspecting Figure 13; it occurs when some options are not available to the players (see Table 10), i.e. when the Utility cannot reduce the investments and Consumers cannot use more HP:

Table 10: Payoffs for the CfD policy, CO2-tax = 200 EUR/tCO2

|  | | Consumers | |
| --- | --- | --- | --- |
|  |  | only HP | some EB |
| Utility | invest more | U- C+ (3) |  |
|  | No invest. | U0 C0 | U0+ C- (2) |

In other words, the NE for the CfD policy instrument is found when Consumers reduce as much as they can their costs by trying to lower their electricity consumption using 100% *HP*. At the same time, the utility makes no investments, so their losses for producing electricity are minimal. *This means both actors are trying not to give any advantage to the other*. This point, which is also the DO in the cooperative game, is not part of the PF, and the NBS has to be found by maximizing the Nash product with respect to the DO (Figure 15 – please note that the NBS is very close to the DO).


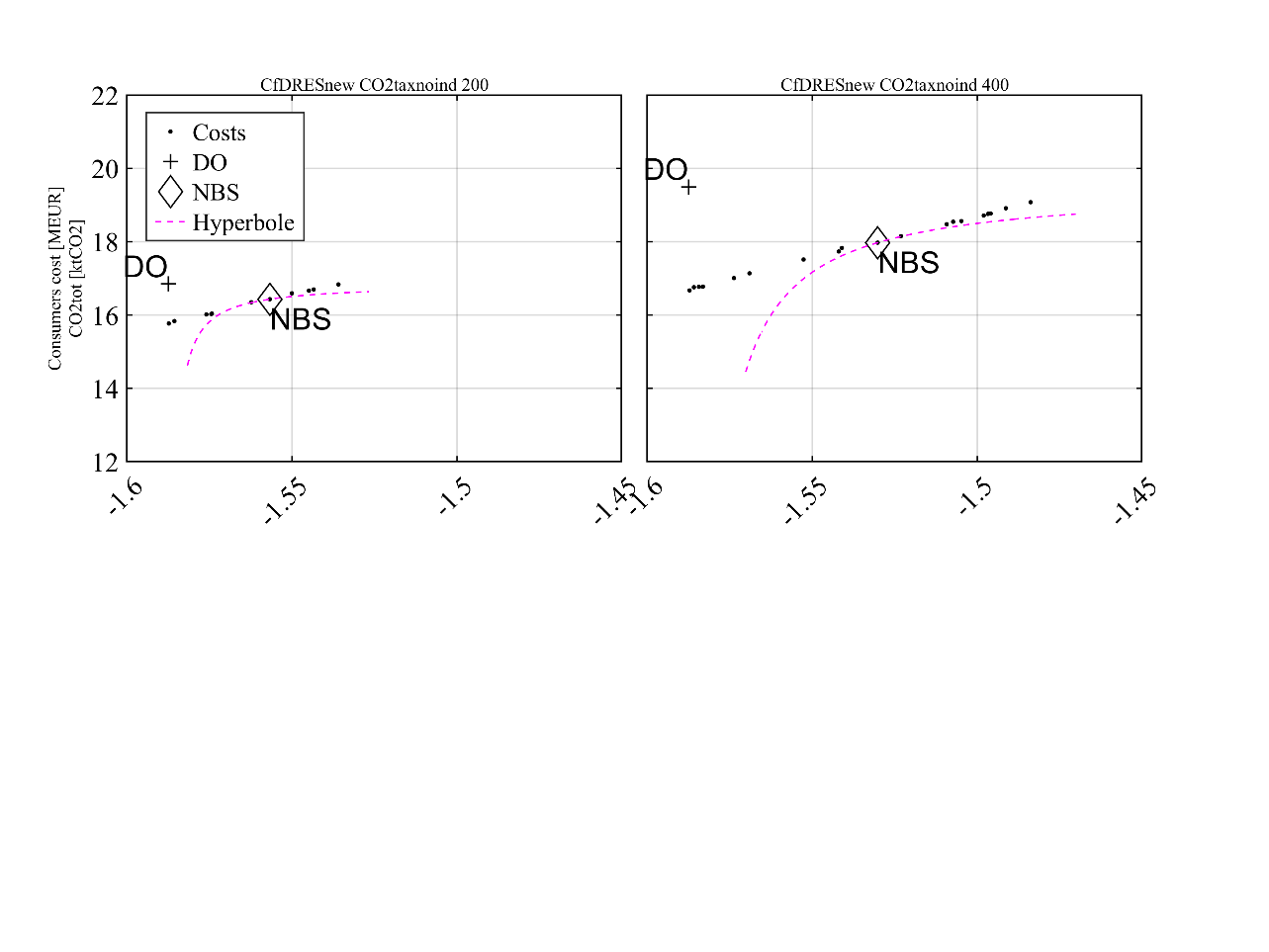


Figure 15: CfD - Disagreement outcome (DO) and Nash bargaining solution (NBS), CO2-tax = 200 and 400 EUR/tCO2.

# Investment subsidies to Consumers

This policy instrument provides government-financed subsidies to Consumers who invest in *EE* measures or the installation of *BioB*. Grant levels of 25%, 50%, and 75% are tested. Results for *BioB* subsidies are presented in Figure 16 and for *EE* in Figure 17. Under the investigated market conditions with an ESP of 25 EUR/MWh, only with grant levels of about 50% (and higher) the *BioB* option or *EE* becomes convenient to the Consumers. Utility losses increase with reduced electricity demand, while the Consumers experience lower costs. The NE is again the leftmost solution on the PF (also DO and then, being on the PF, NBS), the Utility does not invest and the Consumers act according to the provided subsidies (to *BioB*, to *EE* measures, or both) reducing their costs with investments in the most economically convenient technology.


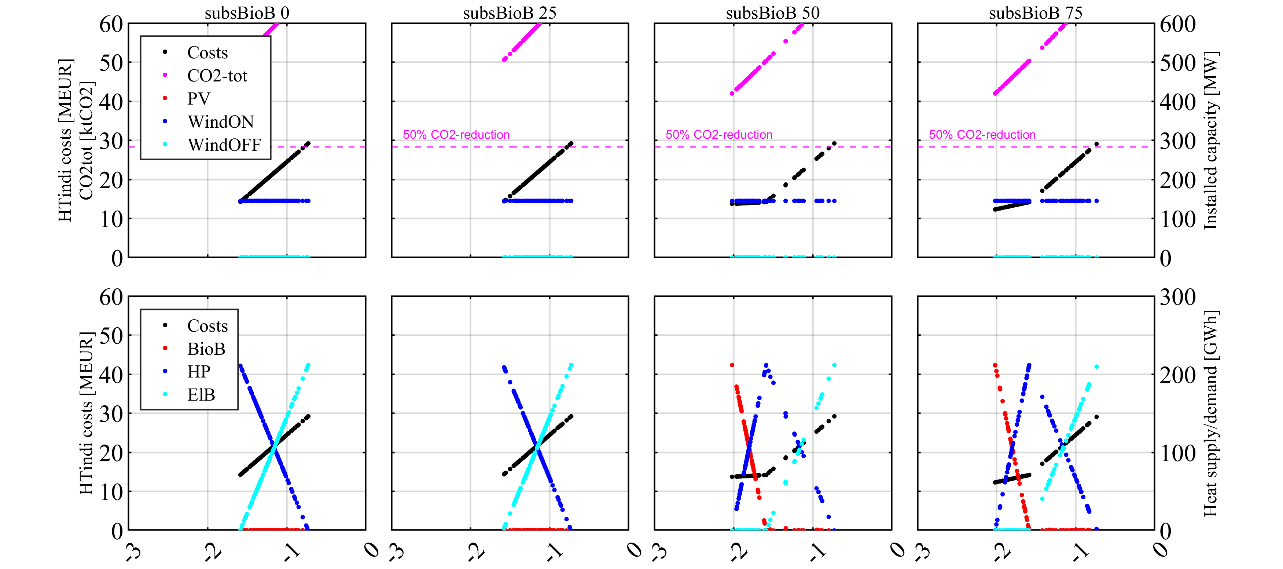


Figure 16: Subsidizing BioB, grant levels: 0, 25, 50, 75%


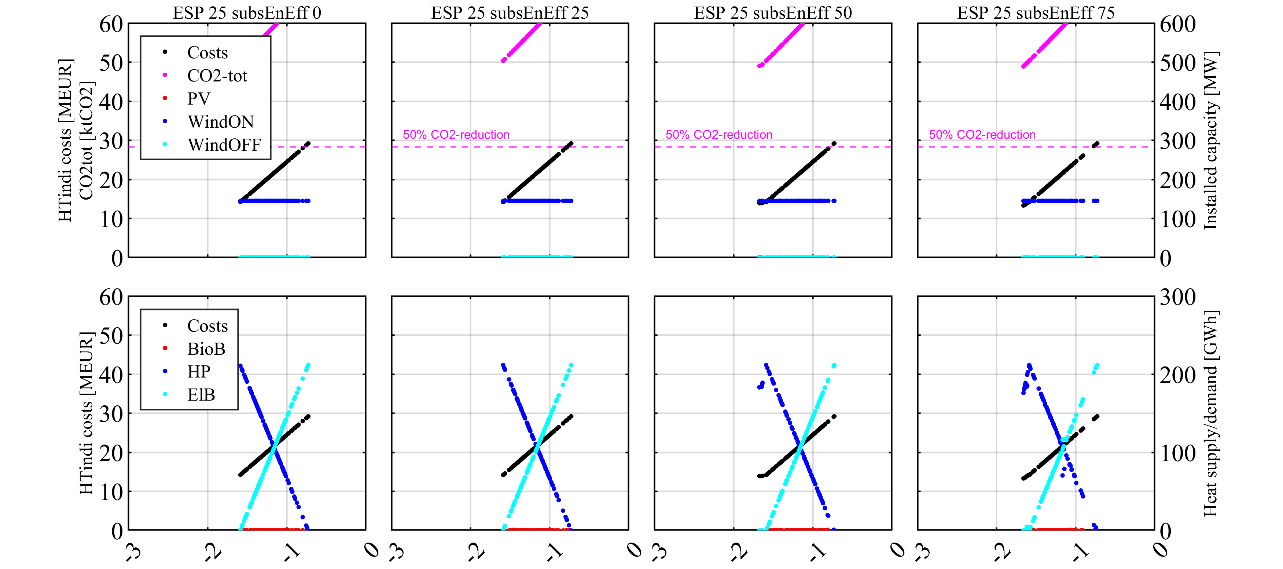


Figure 17: Subsidizing EE measures, grant levels: 0, 25, 50, 75%

# References

[1] OECD. Effective Carbon Rates 2021. 2021.

[2] The World Bank Group. Carbon Pricing Dashboard | Up-to-date overview of carbon pricing initiatives 2022. https://carbonpricingdashboard.worldbank.org/ (accessed May 12, 2022).

[3] Nordhaus WD. Revisiting the social cost of carbon. Proc Natl Acad Sci U S A 2017;114:1518–23. https://doi.org/10.1073/pnas.1609244114.

[4] Swedish Energy Agency. Kvotnivåer 2021. http://www.energimyndigheten.se/fornybart/elcertifikatsystemet/om-elcertifikatsystemet/kvotnivaer/ (accessed July 11, 2022).
